# Adaptive Stimulus Design for Dynamic Recurrent Neural Network Models

**DOI:** 10.3389/fncir.2018.00119

**Published:** 2019-01-22

**Authors:** R. Ozgur Doruk, Kechen Zhang

**Affiliations:** ^1^Department of Electrical and Electronic Engineering, Atilim University, Golbasi, Turkey; ^2^Department of Biomedical Engineering, Johns Hopkins University School of Medicine, Baltimore, MD, United States

**Keywords:** optimal stimulus design, Fisher information matrix, excitatory-inhibitory network, inhomogeneous poisson spike train, maximum likelihood estimation, parameter confounding, Fourier series, sensory coding

## Abstract

We present an adaptive stimulus design method for efficiently estimating the parameters of a dynamic recurrent network model with interacting excitatory and inhibitory neuronal populations. Although stimuli that are optimized for model parameter estimation should, in theory, have advantages over nonadaptive random stimuli, in practice it remains unclear in what way and to what extent an optimal design of time-varying stimuli may actually improve parameter estimation for this common type of recurrent network models. Here we specified the time course of each stimulus by a Fourier series whose amplitudes and phases were determined by maximizing a utility function based on the Fisher information matrix. To facilitate the optimization process, we have derived differential equations that govern the time evolution of the gradients of the utility function with respect to the stimulus parameters. The network parameters were estimated by maximum likelihood from the spike train data generated by an inhomogeneous Poisson process from the continuous network state. The adaptive design process was repeated in a closed loop, alternating between optimal stimulus design and parameter estimation from the updated stimulus-response data. Our results confirmed that, compared with random stimuli, optimally designed stimuli elicited responses with significantly better likelihood values for parameter estimation. Furthermore, all individual parameters, including the time constants and the connection weights, were recovered more accurately by the optimal design method. We also examined how the errors of different parameter estimates were correlated, and proposed heuristic formulas to account for the correlation patterns by an approximate parameter-confounding theory. Our results suggest that although adaptive optimal stimulus design incurs considerable computational cost even for the simplest excitatory-inhibitory recurrent network model, it may potentially help save time in experiments by reducing the number of stimuli needed for network parameter estimation.

## 1. Introduction

One basic problem in systems neuroscience is to understand the relationship between sensory stimulus and neural activity (Simoncelli et al., 2004; Van Hemmen and Sejnowski, 2005). Once the mathematical expression of the stimulus-response relation of a sensory neuron is chosen, all the free parameters of the model can potentially be determined by system identification, or fitting the model to a neurophysiological dataset which consists of the stimuli used and the corresponding elicited neural responses (Westwick and Kearney, 2003; Wu et al., 2006). Accurate parameter estimation requires properly chosen stimuli. For example, stimuli that elicited hardly any responses from the sensory neuron would yield unreliable parameter estimates since the responses reveal little information about the underlying neural system (Christopher deCharms et al., 1998; Wang et al., 2005). Many types of stimuli have been used for model parameter estimation, ranging from random stimuli (Marmarelis and Marmarelis, 1978; Chichilnisky, 2001; Pillow et al., 2008) to natural stimuli (Theunissen et al., 2001). The focus of this paper on automated methods of stimulus design.

In this paper we adopt an adaptive approach to stimulus design to optimize model parameter estimation for a generic excitatory-inhibitory recurrent network model. The adaptive procedure takes into account of the responses to the preceding stimuli, and generates each new stimulus adaptively by maximizing a utility function, which quantifies the usefulness of a given stimulus for model parameter estimation. This approach belongs to a widely used statistical methodology that is often called optimal experimental design, with applications in various disciplines, sometimes under other names (Atkinson and Donev, 1992; Pukelsheim, 1993; Fedorov and Leonov, 2013). The general idea of choosing stimuli adaptively has been explored by many researchers, and various methods have been proposed, with the alopex algorithm as an early example (Harth and Tzanakou, 1974; Benda et al., 2007; DiMattina and Zhang, 2013). Some adaptive methods are not model-based; that is, they do not rely on an explicit model of the stimulus-response relationship, such as the iso-response method (Bölinger and Gollisch, 2012; Gollisch and Herz, 2012; Horwitz and Hass, 2012), stimulus ensemble optimization (Machens et al., 2005), and response maximization by hill-climbing (Nelken et al., 1994; O'Connor et al., 2005) or by genetic algorithms (Bleeck et al., 2003; Yamane et al., 2008; Carlson et al., 2011; Chambers et al., 2014).

The optimal experimental design approach requires an explicit stimulus-response model, and the aim is to estimate the parameters of the given model as accurately as possible. Once all the parameters are determined, the stimulus-response model can potentially predict the responses to all possible stimuli allowed by the model. From an information-theoretic point of view, optimal experimental design based on a suitable model is expected to yield responses that are more informative about the model parameters than responses to nonadaptive random stimuli (Paninski, 2005). For generalized linear models, optimal experimental design method has been shown to be particularly efficient for its parameter estimation, with guaranteed unique solution for Gaussian stimuli (Lewi et al., 2009). Optimal design method can improve parameter estimation for hierarchical feedforward networks or multilayer perceptrons by optimizing the D-optimal metric based on the Fisher information matrix (DiMattina and Zhang, 2011). This method has been used in neurophysiological experiments where sound stimuli with multiple spectral bands were generated adaptively to optimize parameter estimation of the feedforward circuit models of the inferior colliculus (Dekel, 2012; Tam, 2012) and the auditory cortex (Feng et al., 2012; Feng, 2013). The feedforward network models used in these experiments are appropriate for stationary sound stimuli where the emphasis is on spectral features rather than temporal features. Extension to time-varying stimuli would require dynamical neural network models.

Although in this paper we focus on parameter estimation problem only, it is worth mentioning that optimal design can have different goals. One particularly useful example is the optimal design approach that facilitates the comparison of competing models because the best stimuli for model estimation may not be the best stimuli for model discrimination, and vice versa (Sugiyama and Rubens, 2008; Cavagnaro et al., 2010). For example, to compare different feedforward network models, one could combine model estimation and model comparison procedures to generate stimuli that maximally distinguishing the competing models based on the current parameter estimates (DiMattina and Zhang, 2011, 2013). This model comparison method has been successfully applied to auditory neurons recorded from the inferior colliculus in a preliminary neurophysiological experiment where the stimulus design was based on feedforward network models with different numbers of inhibitory neurons (Tam, 2012).

**Figure 1:**
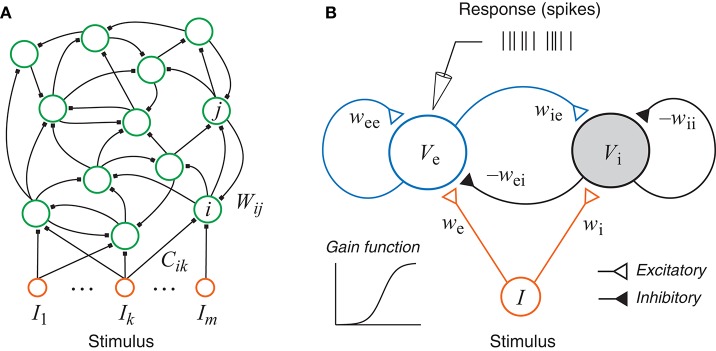
**(A)** A generic recurrent network with external inputs (stimulus). **(B)** A recurrent network with one excitatory unit and one inhibitory unit. Each unit has a sigmoidal gain function and could represent a group of neurons. The responses, as simulated recordings in an experiment, are assumed to be inhomogeneous Poisson spikes based on the continuous rate generated by the state of the excitatory unit.

The neural network model considered in this paper is a simplified recurrent network based on the standard firing rate model (Figure 1). This type of networks has a long history (Amari, 1972; Wilson and Cowan, 1972) and has been used in various research topics (Hopfield, 1984; Beer, 1995; Ledoux and Brunel, 2011; Miller and Fumarola, 2012; Zhang, 2014; Doruk and Zhang, 2018). In particular, such networks have been used to model the neurons in the auditory system (Hancock et al., 1997; Hancock and Voigt, 1999; de la Rocha et al., 2008). Neurophysiological experiments with online adaptive stimulus design for auditory neurons in the inferior colliculus (Dekel, 2012; Tam, 2012) and the auditory cortex (Feng et al., 2012; Feng, 2013) were also based on this type of networks although only spectral information was used while temporal dynamics was ignored.

We seek to extend the optimal design approach to the dynamic recurrent networks with time-varying stimuli. The goal is to estimate the time constants and weight parameters of a given network. The optimal experiment design will be performed by maximizing a metric based on the Fisher information matrix, which is a function of both the stimulus input and the network parameters. As the true network parameters are unknown in real experiments, the Fisher Information matrix is calculated based on the estimated values of the parameters in the current step. An optimization on a time-varying stimulus is not easy and often its parametrization is required. For time-varying inputs such as the auditory stimuli, that can be done by representing the stimuli by a sum of phased cosine elements. We will model the experimental data as extracellular recording of neuronal spike trains, where the only information available is the spiking timing. This situation prevents one to apply traditional parameter estimation techniques such as minimum mean square estimation as it is more suitable for continuous rate data. We will use the likelihood function of the inhomogeneous Poisson spike model whose instantaneous rate is given by the continuous state of a unit. Due to the transient dynamics, we need to derive differential equations to solve for the time courses of the derivatives of the utility function in order to speed up the optimization process. We will examine the advantages as well as the limitations of using adaptive optimal design for dynamic excitatory-inhibitory networks based on the performance of parameter estimation and the computational cost.

## 2. Models and Methods

### 2.1. Continuous Time Recurrent Neural Networks

A generic recurrent neural network may have any number of neurons, and may allow any pattern of connections within the network and any connection weights for the external inputs (Figure 1A). The intrinsic dynamics of each unit is much simpler than conductance-based model (Hodgkin and Huxley, 1952) and resembles the dynamics of the voltage of a passive membrane with a time constant. The output may be interpreted as the firing rate which is related to the voltage by a nonlinear gain function, and the synaptic excitation and inhibition are represented as connection weights between units. The continuous dynamical equation of this generic network can be written as:

(1) $$\begin{array}{l} \tau_{\text{i}}\frac{dV_{\text{i}}}{dt}=-V_{\text{i}}+\underset{j}{\sum }W_{ij}g_{j}\left(V_{j}\right)+\underset{k}{\sum }C_{ik}I_{k} \end{array}$$

where τ_i_ is the time constant of neuron *i* in the network, *V*_i_ is the state of neuron *i* (loosely interpreted as its membrane potential), *W*_*ij*_ is the weight of the synaptic connection from neuron *j* to neuron *i*, the gain function *g*_*j*_ is the input-output function of neuron *j* which transforms its membrane potential to its firing rate, and *C*_*ik*_ is the connection weight from external input *I*_*k*_ to neuron *i*. The gain function is the only source of nonlinearity in Equation (1). A popular choice of the gain function is the logistic sigmoid function given by:

(2) $$\begin{array}{l} g_{j}\left(V_{j}\right)=\frac{\Gamma_{j}}{1+\exp\;\left(-a_{j}\left(V_{j}-h_{j}\right)\right)} \end{array}$$

where Γ_*j*_ is the maximum rate at which neuron *j* can fire, *h*_*j*_ is a soft threshold parameter, and *a*_*j*_ is a slope parameter.

In this paper we focus on the generic excitatory-inhibitory network as shown in Figure 1B. This is the simplest form of the recurrent network described above with only two units, which may be interpreted either as two neurons or as two groups of neurons, one excitatory (e) and one inhibitory (i). The dynamical equations of the system can be written as:

(3) $$\tau_{\text{e}}{\dot{V}}_{\text{e}}=-V_{\text{e}}+w_{\text{ee}}g_{\text{e}}\left(V_{\text{e}}\right)-w_{\text{ei}}g_{\text{i}}\left(V_{\text{i}}\right)+w_{\text{e}}I$$

(4) $$\tau_{\text{i}}\dot{V_{\text{i}}}=-V_{\text{i}}+w_{\text{ie}}g_{\text{e}}\left(V_{\text{e}}\right)-w_{\text{ii}}g_{\text{i}}\left(V_{\text{i}}\right)+w_{\text{i}}I$$

where the subscripts e and i stands for excitatory and inhibitory neurons, respectively, and *I* is a single stimulus variable. One may also interpret *V*_e_ and *V*_i_ as the average activities of populations of excitatory neurons and inhibitory neurons, respectively. Note that in Equations (3) and (4), the weight parameters themselves are assumed to be positive numbers, and their signs are shown explicitly in the equations.

We can rewrite Equations (3) and (4) equivalently as a single equation:

(5) $$\begin{array}{l} \frac{d}{dt}\left[\begin{array}{c} V_{\text{e}}\\ V_{\text{i}} \end{array}\right]=\left[\begin{array}{cc} \beta_{\text{e}} & 0\\ 0 & \beta_{\text{i}} \end{array}\right]\left\{-\left[\begin{array}{c} V_{\text{e}}\\ V_{\text{i}} \end{array}\right]+\left[\begin{array}{cc} w_{\text{ee}} & -w_{\text{ei}}\\ w_{\text{ie}} & -w_{\text{ii}} \end{array}\right]\left[\begin{array}{c} g_{\text{e}}\left(V_{\text{e}}\right)\\ g_{\text{i}}\left(V_{\text{i}}\right) \end{array}\right]+\left[\begin{array}{c} w_{\text{e}}\\ w_{\text{i}} \end{array}\right]I\right\} \end{array}$$

where we define

(6) $$\begin{array}{l} \beta_{\text{e}}=1/\tau_{\text{e}},\;\beta_{\text{i}}=1/\tau_{\text{i}} \end{array}$$

for easier manipulations of the equations. This vector-matrix form is more convenient for mathematical treatment in the following sections.

### 2.2. Inhomogeneous Poisson Spike Model and Likelihood Function

In the model described above, the final output of the excitatory neuron is a continuous firing rate: *r*_e_ = *g*_e_(*V*_e_). Since neuronal spiking activity often has Poisson-like noise (Shadlen and Newsome, 1994), for a better comparison with extracellular neurophysiological recording data, we consider spike trains generated by an inhomogeneous Poisson process, and take *r*_e_ = *g*_e_(*V*_e_) as the instantaneous firing rate (Lewis and Shedler, 1979). We write the rate as *r*_e_(*t*) to emphasize its dependence on time. The probability of finding a spike in the infinitesmal time interval [*t, t* + *dt*) is equal to *r*_e_(*t*)*dt*. Given a spike train (*t*_1_, *t*_2_, …, *t*_*K*_) in the time interval [0, *T*], with any finite *T* > 0, the probability density function for this spike train can be derived from the inhomogeneous Poisson process (Snyder and Miller, 1991; Brown et al., 2002; Eden, 2008), and the result reads:

(7) $$\begin{array}{l} p\left(t_{1},t_{2},\ldots ,t_{K}\right)=\exp\left(-\int_{0}^{T}r_{\text{e}}\left(t\right)dt\right)\overset{K}{\underset{k=1}{\prod }}r_{\text{e}}\left(t_{k}\right) \end{array}$$

This probability density describes how likely a particular spike train (*t*_1_, *t*_2_, …, *t*_*K*_) is generated by the inhomogeneous Poisson process with the rate function *r*_e_(*t*). Of course, this rate function depends implicitly on the network parameters and the stimulus used.

### 2.3. Maximum Likelihood Methods and Parameter Estimation

The network parameters to be estimated are listed below as a vector:

(8) $$\begin{array}{l} \theta =\left[\theta_{1},\ldots ,\theta_{8}\right]=\left[\beta_{\text{e}},\beta_{\text{i}},w_{\text{e}},w_{\text{i}},w_{\text{ee}},w_{\text{ei}},w_{\text{ie}},w_{\text{ii}}\right] \end{array}$$

which includes the time constants and all the connection weights in the excitatory-inhibitory network. Our maximum-likelihood estimation of the network parameters is based on the likelihood function given by Equation (7). It is well known from estimation theory that maximum likelihood estimation is asymptotically efficient; that is, it reaches the Cramér-Rao lower bound for any unbiased estimator in the limit of large data size. Sometimes parameter estimation in optimal design is based on maximum a posteriori estimation (DeGroot, 1970; Pukelsheim, 1993) which is closely linked to the maximum likelihood estimation (Myung, 2003).

It is straightforward to extend the likelihood function in Equation (7) to the situation where there are multiple spike trains elicited by a sequence of stimuli. Suppose there are *M* stimuli and the *m*-th stimulus (*m* = 1, …, *M*) elicits a spike train with a total of *K*_*m*_ spikes in the time window [0, *T*], and the spike timings are given by $S_{m}=\left(t_{1}^{\left(m\right)},t_{2}^{\left(m\right)},\ldots ,t_{K_{m}}^{\left(m\right)}\right)$. By (7), the likelihood function for the spike train *S*_*m*_ is

(9) $$\begin{array}{l} p\left(S_{m}|\theta \right)=\exp\left(-\int_{0}^{T}r_{\text{e}}^{\left(m\right)}\left(t\right)dt\right)\overset{K_{m}}{\underset{k=1}{\prod }}r_{\text{e}}^{\left(m\right)}\left(t_{k}^{\left(m\right)}\right) \end{array}$$

where $r_{\text{e}}^{\left(m\right)}$ is the firing rate in response to the *m*-th stimulus. Note that the rate function $r_{\text{e}}^{\left(m\right)}$ depends implicitly on the network parameters θ and on the stimulus parameters. The left-hand side of (9) emphasizes the dependence on network parameters θ, which is convenient for parameter estimation. The dependence on the stimulus parameters will be discussed in the next section.

We assume that the responses to different stimuli are independent, which is a reasonable assumption when the inter-stimulus intervals are sufficiently large so that any adaptation and plasticity can be ignored. Under this assumption, the overall likelihood function for the collection of all *M* spike trains can be written as

(10) $$\begin{array}{l} L\left(S_{1},\ldots ,S_{M}|\theta \right)=\overset{M}{\underset{m=1}{\prod }}p\left(S_{m}|\theta \right) \end{array}$$

By taking natural logarithm, we obtain the log likelihood function:

(11) $$\begin{array}{l} l\text{ ​}\left(S_{1},\ldots ,S_{M}|\theta \right)=\text{ ln }L\text{​}\left(S_{1},\ldots ,S_{M}|\theta \right)\\ \;=-\sum_{m=1}^{M}\int_{0}^{T}r_{\text{e}}^{\left(m\right)}\text{​}\left(t\right)dt+\sum_{m=1}^{M}\sum_{k=1}^{K_{m}}\text{ ln }\;r_{\text{e}}^{\left(m\right)}\text{​}(t_{k}^{\left(m\right)}) \end{array}$$

Maximum-likelihood estimation of the parameter set is given formally by

(12) $$\begin{array}{l} \hat{\theta }=\arg\underset{\theta }{\text{max}}\;l\left(S_{1},\ldots ,S_{M}|\theta \right) \end{array}$$

Numerical issues related to this optimization problem will be discussed in sections 2.5 and 2.6. In addition, some discussion on the local maxima problems will be provided in section 3.4.

### 2.4. Utility Function of Optimal Design of Stimuli

The optimal design method generates each stimulus by maximizing a utility function, which quantifies the usefulness of a given stimulus for parameter estimation based on the network model. The basic idea is to design stimuli to elicit responses that are most informative about the network parameters. In optimal design method, the utility function *U*(**x**, θ) depends on the stimulus parameters *x*, and typically also on the model parameters θ. An intuitive explanation of the dependence on the model parameters is best illustrated with an example. Suppose we want to estimate a Gaussian tuning curve model with unknown parameters although we may have some idea about the sensible ranges of these parameters. To estimate the height of the tuning curve accurately, we should place a probing stimulus around the likely location of the peak. To estimate the width, the probing stimulus should go to where the tuning curve is likely to have the steepest slope. For the baseline, we should go for the lowest response. This simple example illustrates the fact that an optimally designed stimulus depends on which parameter is to be estimated, and on the prior knowledge of possible parameter values. Since a scalar utility function *U*(**x**, θ) depends on all the parameters θ = [θ_1_, θ_2_, …], optimizing this single scalar function is sufficient to recover all the parameters, at least in theory. Indeed, when a sequence of stimuli are generated by optimal design, the stimuli may alternate spontaneously as if the optimization was performed with respect to each of the parameters one by one (DiMattina and Zhang, 2011). Alternatively, we may also optimize the parameters one by one, as described below.

Once the utility function *U*(**x**, θ) is chosen, the optimally designed stimulus may be written formally as:

(13) $$\begin{array}{l} \hat{\text{x}}=\arg\underset{\text{x}}{\max}U\left(\text{x},\theta \right) \end{array}$$

where the network parameters θ can be obtained by maximum-likelihood estimation from the existing spike data as described in the preceding section. Here the stimulus is specified by vector *x*, which is a set of parameters rather than the actual stimulus itself. Direct computation of the actual time-varying stimulus is not easy because no closed analytical form of the objective function is available and furthermore the computation of the optimal control input generally requires a backward integration or recursion. Instead of struggling with this difficulty, one can restrict the stimulus *I* to a well known natural form such as sum of phased cosines as shown below:

(14) $$\begin{array}{l} I=\overset{N}{\underset{n=1}{\sum }}A_{n}\cos\left(\omega_{n}t+\phi_{n}\right) \end{array}$$

where *A*_*n*_ is the amplitude, ω_*n*_ is the frequency of the *n*-th Fourier component, and ϕ_*n*_ is the phase of the component. We choose a base frequency ω_1_ and set the frequencies of all other components as the harmonics: ω_*n*_ = *nω*_1_ for *n* = 1, …, *N*. Now the stimulus parameters can be summarized by the stimulus parameter vector:

(15) $$\begin{array}{l} \text{x}=\left[x_{1},\cdots \;,x_{2N}\right]=\left[A_{1},\cdots \;,A_{N},\phi_{1},\cdots \;,\phi_{N}\right] \end{array}$$

We sometimes refer to **x** as the stimulus, with it understood that it really means a set of parameters that uniquely specify the actual stimulus *I*.

The Fourier representation of the stimulus in (14) is generic and flexible in the sense that given enough terms, it may potentially approximate any continuous function to arbitrary precision. The optimal design procedure should be able to automatically identify what stimuli are best for recovering the parameters from the observed network state. In general, allowing the stimuli to be time-varying is more powerful than using only stationary stimuli. For example, if we only consider the equilibrium states under stationary stimuli, we always have $\dot{V_{\text{e}}}=\dot{V_{\text{i}}}=0$ so that the time constants τ_e_ and τ_i_ effectively disappear from Equations (3) and (4). In other words, the time constants cannot be recovered solely from the equilibrium states of the networks under stationary stimuli. Some transient responses to time-varying stimuli are required to recover these parameters. In this paper we use the Fourier form as a convenient choice although other representations are also possible.

Some popular choices of the objective function are based on the Fisher information matrix, which is generally defined as:

(16) $$F_{ij}\left(x,\theta \right)=\langle \frac{\partial \text{ ln }p(r|x,\theta )}{\partial \theta_{\text{i}}}\frac{\partial \text{ ln }p(r|x,\theta )}{\partial \theta_{j}}\rangle$$

where *p*(*r*∣**x**, θ) is the probability distribution of the response *r* (number of spikes) elicited by a given stimulus **x** within a certain time window, and the average < > is over all possible responses **r** according to the probability distribution *p*(*r*∣**x**, θ) for fixed **x** and θ. The Fisher information matrix reflects the amount of information contained in the noisy response **r** about the model parameters θ, assuming a generative model given by the conditional probability *p*(**r**∣**x**, θ). So the stimulus designed by maximizing a certain measure of the Fisher information matrix (16) is expected to decrease the error of the estimation of the parameters θ.

Next we give an explicit expression for the response likelihood function *p*(**r**∣**x**, θ), which is different from the spike train likelihood function such as *p*(*t*_1_, *t*_2_, …, *t*_*K*_) in (7) and *p*(*S*_*m*_ ∣ θ) in (9) although they all follow from the same inhomogeneous Poisson model. In our network model, the recorded spike train has an inhomogeneous Poisson distribution with the rate function *r*_e_(*t*). We write this rate as *r*_e_(*t*, **x**, θ) to emphasize its dependence on the stimulus **x** and the network parameters θ. Assuming a small time window of duration Δ*t* centered at time *t*, the response (number of spikes) obeys the Poisson distribution:

(17) $$\begin{array}{l} p\left(r|\text{x},\theta \right)=\frac{\lambda {\left(t,\text{x},\theta \right)}^{r}}{r!}\exp\left(-\lambda \left(t,\text{x},\theta \right)\right) \end{array}$$

where the mean response is given by

(18) $$\begin{array}{l} \lambda \left(t,\text{x},\theta \right)=r_{\text{e}}\left(t,\text{x},\theta \right)\Delta t \end{array}$$

Now the entry of the Fisher information matrix in (16) becomes

(19) $$\begin{array}{l} F_{ij}\left(t,\text{x},\theta \right)=\frac{\Delta t}{r_{\text{e}}\left(t,\text{x},\theta \right)}\frac{\partial r_{\text{e}}\left(t,\text{x},\theta \right)}{\partial \theta_{i}}\frac{\partial r_{\text{e}}\left(t,\text{x},\theta \right)}{\partial \theta_{j}} \end{array}$$

where *t* is added as a variable of *F*_*ij*_ to emphasize its dependence on time.

The utility function *U* can be chosen as a scalar function of the Fisher information matrix **F**. A popular and theoretically well founded choice is the D-optimal design with the utility function:

(20) $$\begin{array}{l} U\left(\text{x},\theta \right)=\det\text{F}\left(\text{x},\theta \right) \end{array}$$

which reflects the inverse volume of the error covariance ellipsoid for all the parameters of the model. One drawback of the method is that the determinant of the Fisher information matrix is not always easy to optimize. The A-optimal design, based on the trace of the Fisher information matrix, is much easier to optimize:

(21) $$\begin{array}{l} U\left(\text{x},\theta \right)=\text{tr}\text{F}\left(\text{x},\theta \right) \end{array}$$

Another alternative is the E-optimal design where the objective function is the smallest eigenvalue of the Fisher information matrix.

In this paper the A-optimality measure of the information matrix is preferred. There is an obvious reason for this preference. As the computational complexity of the optimization algorithms are expected to be high, the necessity of numerical derivative computation should be avoided as much as possible. Since it is not easy to evaluate the derivatives of the eigenvalues and determinants by any means other than numerical approximations it will be convenient to apply a criterion like A-optimality which is simply a the sum of the diagonal elements. Since the Poisson rate function *r*_e_ varies with time, the A-optimal utility function in (21) should be modified by including an integration over time:

(22) $$U\left(x,\theta \right)=\int_{0}^{T}\text{tr}F(t,x,\theta )dt=\int_{0}^{T}\sum_{k=1}^{8}\frac{1}{r_{\text{e}}(t,x,\theta )}{\left(\frac{\partial r_{\text{e}}(t,x,\theta )}{\partial \theta_{k}}\right)}^{2}dt\;$$

where 8 is the total number of parameters (θ_1_, ⋯ , θ_8_), and the time window Δ*t* is ignored because it is a constant coefficient that does not affect the result of the optimization.

For convenience, we can also define the objective function with respect to a single parameter θ_*k*_ as:

(23) $$\begin{array}{l} U_{k}\left(\text{x},\theta \right)=\int_{0}^{T}\frac{1}{r_{\text{e}}\left(t,\text{x},\theta \right)}{\left(\frac{\partial r_{\text{e}}\left(t,\text{x},\theta \right)}{\partial \theta_{k}}\right)}^{2}dt \end{array}$$

The objective function in (22) is identical to

(24) $$\begin{array}{l} U\left(\text{x},\theta \right)=\overset{8}{\underset{k=1}{\sum }}U_{k}\left(\text{x},\theta \right) \end{array}$$

where 8 is the total number of parameters in (8).

The optimization of the D-optimal criterion in (20) is not affected by parameter rescaling, or changing the units of parameters. For example, changing the unit of parameter θ_1_ (say, from msec^−1^ to sec^−1^) is equivalent to rescaling the parameter by a constant coefficient so that θ_1_ → *cθ*_1_. The effect of this transformation is equivalent to a rescaling of the determinant of the Fisher information matrix by a constant factor, namely, det**F** → (det**F**)/*c*^16^, which does not affect the location of the maximum of (20). By contrast, the criterion function in (21) or (22) are affected by parameter rescaling. A parameter with a smaller unit would tend to have larger derivative value and therefore contribute more to (22) than a parameter with a large unit.

To alleviate this scaling problem, we use *U*_*k*_ one by one to generate the stimuli. That is, stimulus 1 is generated by maximizing *U*_1_, and stimulus 2 is generated by maximizing *U*_2_, and so on. Once the 8th stimulus is generated by maximizing *U*_8_, we go back and use *U*_1_ to generate the next stimulus, and so on. Finally, an alternative way to get rid of scale dependence is to introduce logarithm and use $U=\underset{k}{\sum }\text{ ln }U_{k}$ as the criterion, which, however, may become degenerate when *U*_*k*_ approaches 0.

### 2.5. Gradient Computation by Solving Differential Equations

Gradient computation often helps speed up optimization algorithms. The main difficulty of computing gradients in a dynamic network model is the lack of closed form expressions like in the case of static nonlinear mapping given by a multilayer perceptron. Although gradient computations can be easily performed numerically on an explicit function by testing its values in a small neighborhood, this approach is not suitable for our implicit functions because our variables are all governed by differential equations and the increments in perturbation may not be compatible with the differential equations. One feasible remedy is to directly solve for the gradients in self-contained differential equations derived from the original equations (Flila et al., 2010; Telen et al., 2012a,b). We will adapt this method for our excitatory-inhibitory network.

We have two optimization problems in this paper, namely, optimizing the utility function for stimulus design, and optimizing the likelihood function for parameter estimation. To compute the gradients in these two cases, we need to first evaluate the gradients of the network state variable with respect to either the stimulus parameters or the network parameters. We will derive the differential equations satisfied by these gradients by taking derivatives on both sides the original dynamical Equation (5).

First we consider the gradients with respect to the stimulus parameters, which include the amplitudes *A*_*n*_ and the phases ϕ_*n*_ of the Fourier series in Equation (14). We write the stimulus parameter as a vector:

(25) $$\begin{array}{l} \text{x}=\left[x_{1},x_{2},\ldots ,x_{2N}\right]=\left[A_{1},\ldots ,A_{N},\phi_{1},\ldots ,\phi_{N}\right] \end{array}$$

We write the state of the network also as a vector: $\text{v}={\left[V_{\text{e}},V_{\text{i}}\right]}^{\text{T}}$. We take derivatives such as ∂**v**/∂**x** as an independent variable, and solve it directly from the differential equation derived from the original dynamical Equation (5). Taking derivative with respect to **x** on both sides of Equation (5), we obtain the desired differential equation:

(26) $$\begin{array}{l} \frac{d}{dt}\frac{\partial v}{\partial x}=\left[\begin{array}{cc} \beta_{\text{e}} & 0\\ 0 & \beta_{\text{i}} \end{array}\right]\;\{-\frac{\partial v}{\partial x}\\ \;+\left[\begin{array}{cc} w_{\text{ee}} & -w_{\text{ei}}\\ w_{\text{ie}} & -w_{\text{ii}} \end{array}\right]\left[\begin{array}{cc} {g^{\prime }}_{\text{e}}\left(V_{\text{e}}\right) & 0\\ 0 & {g^{\prime }}_{\text{i}}\left(V_{\text{i}}\right) \end{array}\right]\frac{\partial v}{\partial x}+\left[\begin{array}{c} w_{\text{e}}\\ w_{\text{i}} \end{array}\right]\frac{\partial I}{\partial x}\} \end{array}$$

where $g_{\text{e}}^{\prime }$ and $g_{\text{i}}^{\prime }$ are the derivatives of the gain functions *g*_e_ and *g*_i_, respectively, and the matrices derivatives are defined in the usual manner:

(27) $$\begin{array}{l} \frac{\partial I}{\partial x}=\left[\begin{array}{cccccc} \frac{\partial I}{\partial A_{1}} & \ldots & \frac{\partial I}{\partial A_{N}}, & \frac{\partial I}{\partial \phi_{1}} & \ldots & \frac{\partial I}{\partial \phi_{N}} \end{array}\right] \end{array}$$

and

(28) $$\begin{array}{l} \frac{\partial v}{\partial x}=\left[\begin{array}{cccccc} \frac{\partial V_{\text{e}}}{\partial A_{1}} & \ldots & \frac{\partial V_{\text{e}}}{\partial A_{N}} & \frac{\partial V_{\text{e}}}{\partial \phi_{1}} & \ldots & \frac{\partial V_{\text{e}}}{\partial \phi_{N}}\\ \frac{\partial V_{\text{i}}}{\partial A_{1}} & \ldots & \frac{\partial V_{\text{i}}}{\partial A_{N}} & \frac{\partial V_{\text{i}}}{\partial \phi_{1}} & \ldots & \frac{\partial V_{\text{i}}}{\partial \phi_{N}} \end{array}\right] \end{array}$$

Equation (26) can be written equivalently in the shorthand form:

(29) $$\begin{array}{l} \frac{d}{dt}\frac{\partial v}{\partial x}=\text{B }\left\{-\frac{\partial v}{\partial x}+\text{W}\text{G}\frac{\partial v}{\partial x}+\text{w}\frac{\partial I}{\partial x}\right\} \end{array}$$

where $B=\left[\begin{array}{cc} \beta_{\text{e}} & 0\\ 0 & \beta_{\text{i}} \end{array}\right],W=\left[\begin{array}{cc} w_{\text{ee}} & -w_{\text{ei}}\\ w_{\text{ie}} & -w_{\text{ii}} \end{array}\right],\;G=\left[\begin{array}{cc} {g^{\prime }}_{\text{e}}(V_{\text{e}}) & 0\\ 0 & {g^{\prime }}_{\text{i}}(V_{\text{i}}) \end{array}\right],$ and $w=\left[\begin{array}{c} w_{\text{e}}\\ w_{\text{i}} \end{array}\right]$

Next we consider the gradients with respect to the network parameters: θ = [θ_1_, …, θ_8_] = [β_e_, β_i_, *w*_e_, *w*_i_, *w*_ee_, *w*_ei_, *w*_ie_, *w*_ii_]. By taking derivative with respect to θ_*k*_ on both sides of Equation (5), we obtain

(30) $$\begin{array}{l} \frac{d}{dt}\frac{\partial v}{\partial \theta_{k}}=\text{B }\left\{-\frac{\partial v}{\partial \theta_{k}}+\text{W}\text{G}\frac{\partial v}{\partial \theta_{k}}\right\}+{\text{z}}_{k} \end{array}$$

where $\frac{\partial v}{\partial \theta_{k}}={\left[\frac{\partial V_{\text{e}}}{\partial \theta_{k}},\frac{\partial V_{\text{i}}}{\partial \theta_{k}}\right]}^{\text{T}}$ and the last term **z**_*k*_ refers to the extra components resulting from the chain rule of differentiation. These extra terms are presented in Table 1.

**Table 1:** The extra components *z*_*k*_ in Equation (30).

**k**	**Parameter θ_*k*_**	**Extra term z_*k*_ in Equation (30)**
1	β_e_	$\left[\begin{array}{cc} 1 & 0\\ 0 & 0 \end{array}\right]\left\{-\left[\begin{array}{c} V_{\text{e}}\\ V_{\text{i}} \end{array}\right]+w\left[\begin{array}{c} g_{\text{e}}\left(V_{\text{e}}\right)\\ g_{\text{i}}\left(V_{\text{i}}\right) \end{array}\right]+wI\right\}$
2	β_i_	$\left[\begin{array}{cc} 0 & 0\\ 0 & 1 \end{array}\right]\left\{-\left[\begin{array}{c} V_{\text{e}}\\ V_{\text{i}} \end{array}\right]+w\left[\begin{array}{c} g_{\text{e}}\left(V_{\text{e}}\right)\\ g_{\text{i}}\left(V_{\text{i}}\right) \end{array}\right]+wI\right\}$
3	*w*_e_	$B\left[\begin{array}{c} 1\\ 0 \end{array}\right]I$
4	*w*_i_	$B\left[\begin{array}{c} 0\\ 1 \end{array}\right]I$
5	*w*_ee_	$B\left[\begin{array}{cc} 1 & 0\\ 0 & 0 \end{array}\right]\left[\begin{array}{c} g_{\text{e}}\left(V_{\text{e}}\right)\\ g_{\text{i}}\left(V_{\text{i}}\right) \end{array}\right]$
6	*w*_ei_	$B\left[\begin{array}{cc} 0 & -1\\ 0 & 0 \end{array}\right]\left[\begin{array}{c} g_{\text{e}}\left(V_{\text{e}}\right)\\ g_{\text{i}}\left(V_{\text{i}}\right) \end{array}\right]$
7	*w*_ie_	$B\left[\begin{array}{cc} 0 & 0\\ 1 & 0 \end{array}\right]\left[\begin{array}{c} g_{\text{e}}\left(V_{\text{e}}\right)\\ g_{\text{i}}\left(V_{\text{i}}\right) \end{array}\right]$
8	*w*_ii_	$B\left[\begin{array}{cc} 0 & 0\\ 0 & -1 \end{array}\right]\left[\begin{array}{c} g_{\text{e}}\left(V_{\text{e}}\right)\\ g_{\text{i}}\left(V_{\text{i}}\right) \end{array}\right]$

We also need to consider the second-order cross derivatives as needed for maximizing the trace of the Fisher information matrix. Taking derivative of (29) with respect to θ_*k*_, we find:

(31) $$\frac{d}{dt}\frac{\partial^{2}v}{\partial x\partial \theta_{k}}=B\left\{-\frac{\partial^{2}v}{\partial x\partial \theta_{k}}+WG\frac{\partial^{2}v}{\partial x\partial \theta_{k}}+WG^{\prime }\text{diag​}\left(\frac{\partial v}{\partial \theta_{k}}\right)\frac{\partial v}{\partial x}\right\}+Z_{k}$$

where ${\text{G}}^{\prime }=\left[\begin{array}{cc} g_{\text{e}}^{\prime \prime }\left(V_{\text{e}}\right) & 0\\ 0 & g_{\text{i}}^{\prime \prime }\left(V_{\text{i}}\right) \end{array}\right]$, $\text{diag}\left(\frac{\partial v}{\partial \theta_{k}}\right)=\left[\begin{array}{cc} \frac{\partial V_{\text{e}}}{\partial \theta_{k}} & 0\\ 0 & \frac{\partial V_{\text{i}}}{\partial \theta_{k}} \end{array}\right]$, and

(32) $$\begin{array}{l} \frac{\partial^{2}v}{\partial \text{x}\partial \theta_{k}}=\left[\begin{array}{cccccc} \frac{\partial^{2}V_{\text{e}}}{\partial A_{1}\partial \theta_{k}} & \ldots & \frac{\partial^{2}V_{\text{e}}}{\partial A_{N}\partial \theta_{k}} & \frac{\partial^{2}V_{\text{e}}}{\partial \phi_{1}\partial \theta_{k}} & \ldots & \frac{\partial^{2}V_{\text{e}}}{\partial \phi_{N}\partial \theta_{k}}\\ \frac{\partial^{2}V_{\text{i}}}{\partial A_{1}\partial \theta_{k}} & \ldots & \frac{\partial^{2}V_{\text{i}}}{\partial A_{N}\partial \theta_{k}} & \frac{\partial^{2}V_{\text{i}}}{\partial \phi_{1}\partial \theta_{k}} & \ldots & \frac{\partial^{2}V_{\text{i}}}{\partial \phi_{N}\partial \theta_{k}} \end{array}\right] \end{array}$$

which is compatible with (28). The last term **Z**_*k*_ is specified in Table 2.

**Table 2:** The extra components *Z*_*k*_ in Equation (31).

**k**	**Parameter θ_*k*_**	**Extra term Z_*k*_ in Equation (31)**
1	β_e_	$\left[\begin{array}{cc} 1 & 0\\ 0 & 0 \end{array}\right]\left\{-\frac{\partial v}{\partial x}+\text{W}\text{G}\frac{\partial v}{\partial x}+w\frac{\partial I}{\partial x}\right\}$
1	β_i_	$\left[\begin{array}{cc} 0 & 0\\ 0 & 1 \end{array}\right]\left\{-\frac{\partial v}{\partial x}+\text{W}\text{G}\frac{\partial v}{\partial x}+w\frac{\partial I}{\partial x}\right\}$
3	*w*_e_	$\text{B}\left[\begin{array}{c} 1\\ 0 \end{array}\right]\frac{\partial I}{\partial x}$
4	*w*_i_	$\text{B}\left[\begin{array}{c} 0\\ 1 \end{array}\right]\frac{\partial I}{\partial x}$
5	*w*_ee_	$\text{B}\left[\begin{array}{cc} 1 & 0\\ 0 & 0 \end{array}\right]G\frac{\partial v}{\partial x}$
6	*w*_ei_	$\text{B}\left[\begin{array}{cc} 0 & -1\\ 0 & 0 \end{array}\right]G\frac{\partial v}{\partial x}$
7	*w*_ie_	$\text{B}\left[\begin{array}{cc} 0 & 0\\ 1 & 0 \end{array}\right]G\frac{\partial v}{\partial x}$
8	*w*_ii_	$\text{B}\left[\begin{array}{cc} 0 & 0\\ 0 & -1 \end{array}\right]G\frac{\partial v}{\partial x}$

Now we are ready to evaluate the derivatives of the mean firing rate *r*_e_ = *g*_e_(*V*_e_) with respect to the network parameters θ_*k*_ in (8) and the stimulus parameters *x*_*j*_ in (25). The first and the second order derivatives are:

(33) $$\begin{array}{l} \frac{\partial r_{\text{e}}}{\partial \theta_{k}}=g_{\text{e}}^{\prime }\left(V_{\text{e}}\right)\frac{\partial V_{\text{e}}}{\partial \theta_{k}} \end{array}$$

(34) $$\begin{array}{l} \frac{\partial r_{\text{e}}}{\partial x_{l}}=g_{\text{e}}^{\prime }\left(V_{\text{e}}\right)\frac{\partial V_{\text{e}}}{\partial x_{l}} \end{array}$$

(35) $$\begin{array}{l} \frac{\partial^{2}r_{\text{e}}}{\partial x_{l}\partial \theta_{k}}=g_{\text{e}}^{\prime \prime }\left(V_{\text{e}}\right)\frac{\partial V_{\text{e}}}{\partial x_{l}}\frac{\partial V_{\text{e}}}{\partial \theta_{k}}+g_{\text{e}}^{\prime }\left(V_{\text{e}}\right)\frac{\partial^{2}V_{\text{e}}}{\partial x_{l}\partial \theta_{k}} \end{array}$$

These formulas are expressed in terms of the derivatives $\frac{\partial V_{\text{e}}}{\partial \theta_{k}}$, $\frac{\partial V_{\text{e}}}{\partial x_{l}}$ and $\frac{\partial^{2}V_{\text{e}}}{\partial x_{l}\partial \theta_{k}}$, which are regarded as independent dynamical variables that can be solved from the three differential Equations (29)–(31). In our simulations, the initial conditions were always assumed to be the equilibrium state, and the initial values of the derivatives were set to zero.

Next, we evaluate the gradient of the utility function in Equation (22) with respect to the stimulus parameters *x*_*l*_ as follows:

(36) $$\begin{array}{ll} \frac{\partial U}{\partial x_{l}} & =\int_{0}^{T}\frac{\partial }{\partial x_{l}}\overset{8}{\underset{k=1}{\sum }}\frac{1}{r_{\text{e}}}{\left(\frac{\partial r_{\text{e}}}{\partial \theta_{k}}\right)}^{2}dt \end{array}$$

(37) $$\begin{array}{l} =\int_{0}^{T}\overset{8}{\underset{k=1}{\sum }}\left\{-\frac{1}{r_{\text{e}}^{2}}\frac{\partial r_{\text{e}}}{\partial x_{l}}{\left(\frac{\partial r_{\text{e}}}{\partial \theta_{k}}\right)}^{2}+\frac{2}{r_{\text{e}}}\frac{\partial r_{\text{e}}}{\partial \theta_{k}}\frac{\partial^{2}r_{\text{e}}}{\partial x_{l}\partial \theta_{k}}\right\}dt \end{array}$$

where the last expression is written in terms of the derivatives that are already evaluated by Equations (33)–(35). If we design the stimulus with respect to one parameter by optimizing the utility function in Equation (23), we rewrite the above by removing the summation and obtain:

(38) $$\begin{array}{l} \frac{\partial U_{k}}{\partial x_{l}}=\int_{0}^{T}\frac{\partial }{\partial x_{l}}\frac{1}{r_{\text{e}}}{\left(\frac{\partial r_{\text{e}}}{\partial \theta_{k}}\right)}^{2}dt \end{array}$$

(39) $$\begin{array}{ll} = & \int_{0}^{T}\left\{-\frac{1}{r_{\text{e}}^{2}}\frac{\partial r_{\text{e}}}{\partial x_{l}}{\left(\frac{\partial r_{\text{e}}}{\partial \theta_{k}}\right)}^{2}+\frac{2}{r_{\text{e}}}\frac{\partial r_{\text{e}}}{\partial \theta_{k}}\frac{\partial^{2}r_{\text{e}}}{\partial x_{l}\partial \theta_{k}}\right\}dt \end{array}$$

Lastly, for maximum likelihood estimation, one needs the gradient of the log likelihood function of spike trains in (11):

(40) $$\frac{\partial l}{\partial \theta_{k}}=-\sum_{m=1}^{M}\int_{0}^{T}\frac{\partial r_{\text{e}}^{\left(m\right)}\text{​}\left(t\right)}{\partial \theta_{k}}dt+\sum_{m=1}^{M}\sum_{k=1}^{K_{m}}\;\frac{1}{r_{\text{e}}^{\left(m\right)}\text{​}\left(t_{k}^{\left(m\right)}\right)}\frac{\partial r_{\text{e}}^{\left(m\right)}\text{​}\left(t_{k}^{\left(m\right)}\right)}{\partial \theta_{k}}$$

The right-hand side is already written in terms of derivatives that can be evaluated by (33).

### 2.6. Practical Numerical Issues Related to Optimization

As described in the preceding sections, our optimal design method requires solving two separate maximization problems as given by Equations (12) and (13). Since the goal of this paper is not to develop or implement optimization algorithms, we used the optimization programs available in MATLAB (R2013b) for all the simulation results reported in the paper. After comparison with several MATLAB optimization programs including genetic algorithms, simulated annealing and pattern search, we found that the function *fmincon*, with the default interior-point method of constrained nonlinear optimization, performed adequately for our problems.

The function *fmincon* and similar algorithms are local optimizers. In order to find a good optimum, the algorithms are often repeated with multiple initial guesses and the best one is chosen according to the value of the objective and gradient value at the termination point. This is especially important in the optimal design part as the utility function in Equation (23) may have lots of local maxima. The local maxima problem also exists for the likelihood function (11), but to a lesser extent. This is because as the number of stimuli increases, the likelihood function tends to converge to the same optimum for different initial guesses. See section 3.4 for a detailed discussion of this issue.

The local optimization algorithms such as *fmincon* need the gradient of the objective function. Although the gradient could be computed numerically, it is better to use the derivatives obtained directly by solving differential equations as explained in the preceding section. This is because numerical gradient computation is much slower and also increases the risk of singularities in the solution.

Finally, MATLAB parallel computation toolbox can speed up the optimization. We obtained a speedup by a factor of 5 even on a PC with a single Intel i7 six-core processor.

### 2.7. Procedural Information

In this section, we will summarize the overall procedure to show how the optimal design and parameter estimation algorithm works together in an automated loop. The first issue is the choice of the initial parameter values. In the beginning of an experiment (or simulation in our case), one usually has no idea about the true values of the network parameter vector θ, although one may have some prior information about plausible or reasonable ranges of acceptable parameter values. Since the optimal design method needs a current parameter estimate, one can assign a randomly chosen initial parameter vector from some prior distribution. In our simulations, we simply drew each parameter randomly from a uniform distribution between a lower bound and an upper bound of acceptable parameter values. One can find the details about the parametric bounds in Table 3. The stimulus amplitude coefficients [*A*_*n*_ in (14)] also need to be bounded because in the real world the total energy of the sound stimulus is finite. In simulations, very large amplitudes may lead to unrealistically large responses and instabilities which may break the optimal design procedures. An upper bound on *A*_*n*_'s can be found by a few initial simulations, and if no instabilities are detected one will be fine with the decided value of the bound.

**Table 3:** True values, bounds, and estimates of network parameters.

**Parameter**	**True value θ**	**Lower bound θ**_**min**_	**Upper bound θ**_**max**_	**Estimate ${\hat{\theta }}_{opt}$ (mean±std)**	**Estimate ${\hat{\theta }}_{rand}$ (mean±std)**
β_e_	50	0	100	49.94 ± 0.86	50.07 ± 1.63
β_i_	25	0	100	25.18 ± 1.44	25.23 ± 1.96
*w*_e_	1	0	2	1.002 ± 0.036	0.998 ± 0.039
*w*_i_	0.7	0	2	0.698 ± 0.068	0.715 ± 0.100
*w*_ee_	1.2	0	3	1.227 ± 0.069	1.247 ± 0.107
*w*_ei_	2	0	3	2.069 ± 0.187	2.071 ± 0.227
*w*_ie_	0.7	0	3	0.712 ± 0.095	0.784 ± 0.171
*w*_ii_	0.4	0	3	0.456 ± 0.199	0.569 ± 0.374

*The true values of the 8 parameters in the excitatory-inhibitory network in Equation (5) are shown, followed by the lower and upper bounds used during the maximum-likelihood optimization process. The means and the standard deviations of the maximum-likelihood estimates ${\hat{\theta }}_{\text{opt}}$ and ${\hat{\theta }}_{\text{rand}}$ were obtained from 100 repeated trials, where each trial had 120 optimally designed stimuli and 120 random stimuli, respectively*.

The overall process of the algorithm is summarized below:

1. Set *i* = 1 (iteration count)
2. Set the current estimate $\hat{\theta }$ as a random vector between θ_min_ and θ_max_ (from Table 3)
3. Set *k* = 1 (parameter count)
4. Optimize $U_{k}\left(\text{x},\hat{\theta }\right)$ (as in Equation 23) to generate stimulus $\hat{\text{x}}$, which elicits a new response.
5. Update the current maximum likelihood estimate, $\hat{\theta }$, by including the new response data.
6. Set *k* → *k* + 1. If *k* > 8 (total number of parameters) set *i* → *i* + 1 and go to the next step, otherwise go to **Step 4**.
7. If *i* > *N*_itr_ stop and report the result as $\hat{\theta }$, otherwise go to **Step 3**

Here *N*_itr_ is the total number of iterations. The total number of stimuli generated by the procedure is equal to 8*N*_itr_. The optimal design results in the next section were all obtained by this procedure.

## 3. Simulation Results

In this section, we will summarize our computer simulation results based on the adaptive stimulus design and network parameter estimation algorithms as described in the preceding section. Besides the properties of the optimally designed stimuli and the accuracy of the parameter estimation, we will also examine how the errors of different parameter estimates are correlated, and how the correlations might be accounted for by parameter confounding formulas derived from the network dynamical equations.

### 3.1. Details of the Example Problem

All simulations of the excitatory-inhibitory network (Figure 1B and Equation 5) were based on the generating values (sometimes also referred to as “true values”) of the parameters given in Table 3. The gain functions *g*_e_(*V*_e_) and *g*_i_(*V*_i_) require additional parameters, namely, Γ_e_, *a*_e_, *h*_e_, Γ_i_, *a*_i_, and *h*_i_. These gain functions are given by Equation (2) except that the subscript *j* should be replaced by either ‘e’ or ‘i’. Because this research was aimed at the estimation of the network parameters only, the gain functions, representing the input-output properties of individual units, were assumed to be fixed with the parameter values Γ_e_ = 100, *a*_e_ = 0.04, *h*_e_ = 70, Γ_i_ = 50, *a*_i_ = 0.04, and *h*_i_ = 35. This assumption simplifies the parameter estimation problem because now we can focus on network parameter estimation only. This assumption is not absolutely required because our method could be readily extended to estimate these parameters as well. The simplification here is equivalent to assuming that we already know the input-output relations of each individual unit. In realistic situation, we may use the average input-output relationship of each neuronal type if it is known because each unit in the model could correspond to a subpopulation of neurons as describe before.

**Figure 2:**
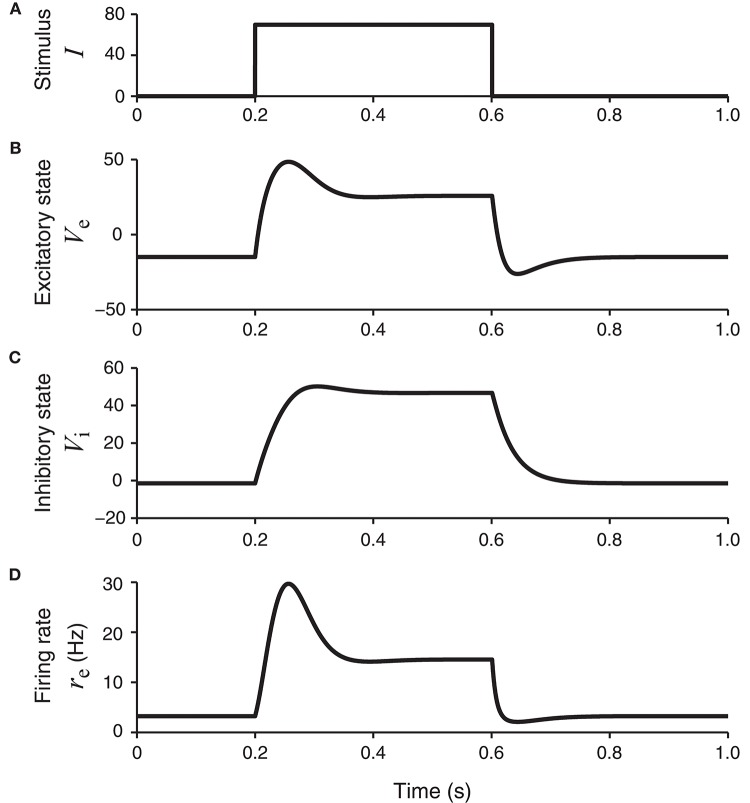
The network model in Figure 1B in response to a square-wave stimulus. The states of the excitatory and inhibitory units, *V*_e_ and *V*_i_, are shown, together with the continuous firing rate of the excitatory unit, *r*_e_ = *g*_e_ (*V*_e_). The firing rate of the excitatory unit (bottom panel) has a transient component with higher firing rates, followed by a plateau or sustained component with lower firing rates.

This set of parameters allows the network to have a unique equilibrium state for each stationary input. For a square wave stimulus (Figure 2A), the resultant excitatory and inhibitory neural membrane potential responses, namely, *V*_e_(*t*) and *V*_i_(*t*), show both a transient component (initial peak) and a sustained component (later plateau) (Figures 2B,C). The peak response of the inhibitory unit rises more slowly than the excitatory unit. The peak of the excitatory unit is more pronounced, which is especially clear in the firing rate *r*_e_(*t*) = *g*_e_(*V*_e_(*t*)) as shown in Figure 2D.

The optimization of the stimuli requires that the maximum power level in a single stimulus is bounded. This is a precaution to protect the model from potential instabilities due to over-stimulation. In real experiments, the stimuli should also have bounded maximum energy and subject will also need to be protected from over-stimulations. Consider the Fourier series for the stimulus in (14). As the amplitude parameter is assumed to be nonnegative (*A*_*n*_ ≥ 0), assigning an upper bound defined as *A*_max_ should be enough. This is applied to all stimulus amplitudes *A*_*n*_. In this research, a fixed setting of *A*_max_ = 120 is chosen. The lower bound is obviously *A*_min_ = 0. The phase parameter ϕ_*n*_ is allowed to vary freely without any lower or upper bounds as the cosine function itself is already bounded (−1 ≤ cos ≤ 1). The frequency ω_*n*_ of the stimulus component is the *n*-th harmonics of a base frequency *f*_*base*_; that is, ω_*n*_ = 2π*nf*_*base*_. Since we assumed a simulation time of *T*_*opt*_ = 3 s, we made a reasonable choice of the base frequency as $f_{base}=\frac{10}{3}$ Hz or 3.33 Hz. So we have chosen an integer relationship between the stimulation frequency and simulation time. The number of stimulus components *N* is chosen as *N* = 5 which is found to be reasonable concerning speed and performance balance.

Optimization algorithms such as *fmincon* requires an initial guess of the optimum solution. To make the initial choice uninformative, we picked each initial value randomly with a uniform distribution between the lower bound and the upper bound. In the optimization of stimuli, the initial amplitudes were uniformly distributed between [0, *A*_max_] and phases were uniformly distributed between [−π, π). Although we did not have any constraints on the phase parameter, we limited the initial phase values to a safe assumed range. We follow a similar strategy for the network parameter estimation based on maximum likelihood method. The multiple initial guesses were chosen randomly from a set of values uniformly distributed between the lower and upper bounds defined in Table 3.

In section 2.3, one recall from (10) or (11) that the likelihood estimation should produce better results when the number of spike train samples, *M*, increases. Because of this fact, the likelihood function should always be evaluated with all the available spike trains generated since the beginning of the simulated trial. The total number of spike trains, *M*, is always equal to the total number of stimuli. If the optimal design process is iterated *N*_itr_ times, one will have *M* stimuli with *M* = 8*N*_itr_ due to the fact that each iteration has 8 optimal designs sub-steps for each of the eight parameters in (8) (see section 2.4). In our simulations, we typically used 15 iterations *N*_itr_ = 15, so that we had *M* = 120 stimuli and the likelihood in (11) had 120 samples. This also means that optimal design and subsequent parameter estimation were iterated 120 times for each simulated trial. To obtain the statistics of the estimates, the trial was repeated 100 times for each case.

### 3.2. Statistics of Optimally Designed Stimuli

**Figure 3:**
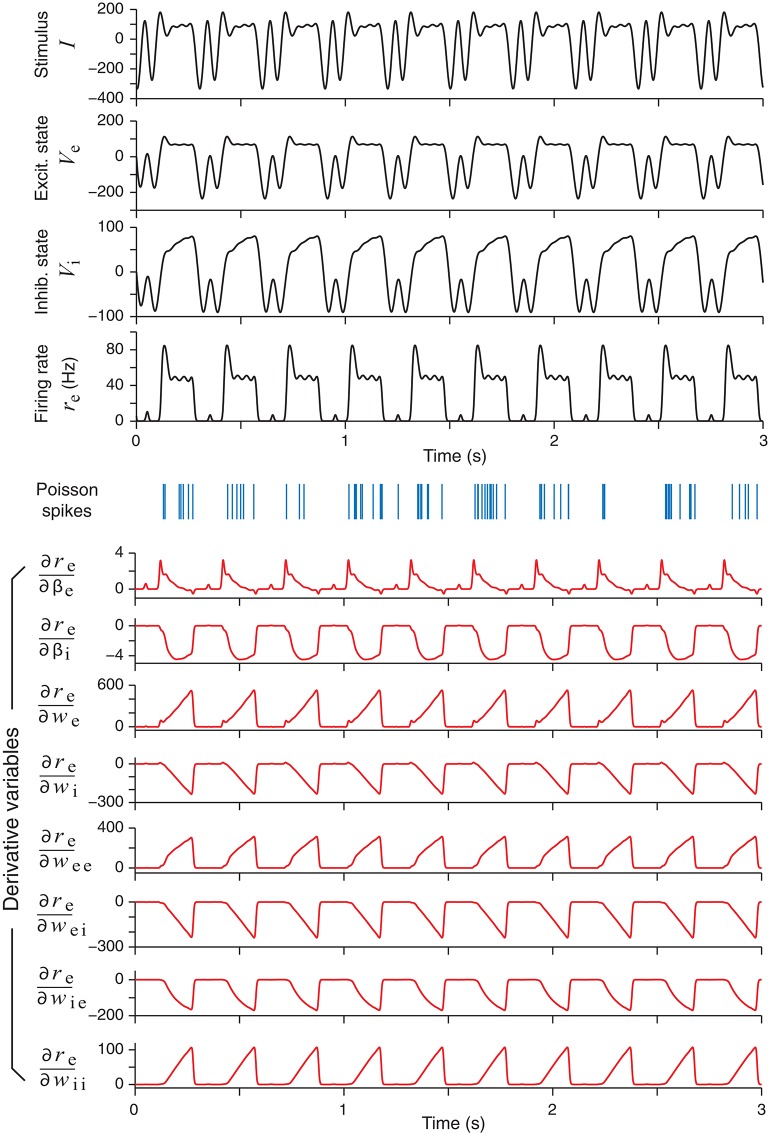
An example of an optimally designed stimulus with a duration of 3 s (top panel). The responses of the excitatory and the inhibitory units in the network are shown below, followed by an example of spike trains (blue vertical bars) generated by an inhomogeneous Poisson process according to the continuous firing rate of the excitatory unit (*r*_e_). In response to this stimulus, the time courses of eight derivatives variables, namely, the derivatives of the firing rate *r*_e_ with respect to all the network parameters, are shown as red curves. These derivatives were solved directly from the differential Equation (29).

With all the necessary information from section 3.1, one can perform an optimal design experiment where stimuli are generated adaptively to elicit neural responses. An example of an optimally design stimulus, together with the elicited responses, is shown in Figure 3. It is noted that the optimally designed stimulus in the top panel of Figure 3 is periodic in time because it is modeled by the Fourier series in (14).

In addition to the network responses, the bottom half of Figure 3 shows the time courses of the parametric sensitivity derivatives $\frac{\partial r_{\text{e}}}{\partial \theta_{i}}$ which are generated by integrating (30) and then substituting to (33). The sensitivity derivatives varied greatly in their magnitudes or maximum absolute values. In Figure 3, the magnitudes of the derivatives with respect to the weight parameters (*w*_e_, *w*_ei_, etc.) were more than 10 times that of the time parameters (β_e_ and β_i_). In particular, the maximum value of $\frac{\partial r_{\text{e}}}{\partial w_{\text{e}}}$ was over 100 times greater than that of $\frac{\partial r_{\text{e}}}{\partial \beta_{\text{e}}}$. This difference supports the idea that optimizing the Fisher Information Metric in (23) should be performed with respect to each single parameter separately as described at the end of section 2.4. Otherwise, if one only optimizes the sum in (24) as a whole, then a term with a low-sensitivity parameter could be easily overwhelmed by the fluctuations of a term with a high-sensitivity parameter. We also note that the times courses of some of the sensitivity derivatives looked very similar, while others looked like mirror images of one another. This means that small increments of these parameters affect the output firing rate *r*_e_ in either the same direction or the opposite direction, due to the intrinsic dynamics of the network and how the excitatory unit and inhibitory unit are wired up.

**Figure 4:**
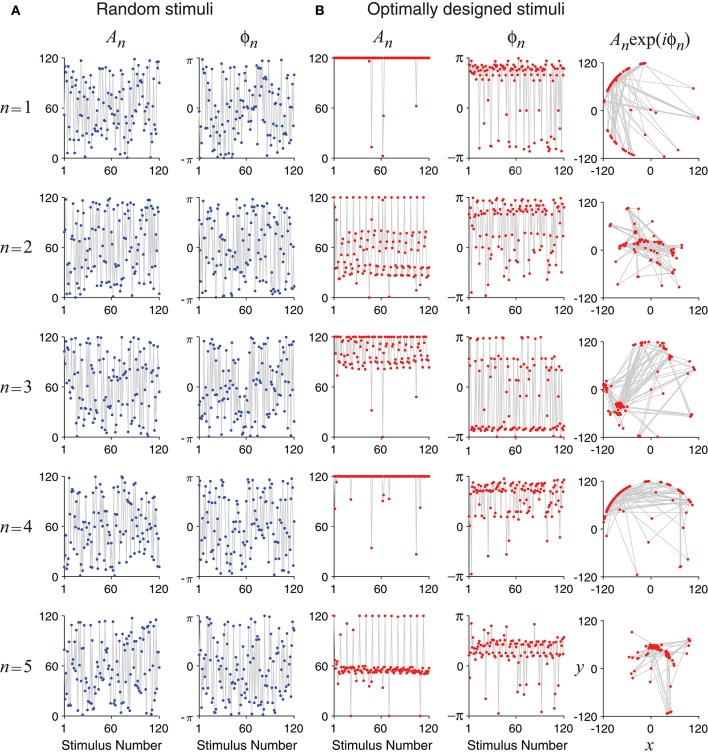
Time evolution of the optimally designed stimuli in a simulated experiment with 120 iterations. **(A)** Random stimuli with uniformly distributed random Fourier amplitudes *A*_*n*_ and phases ϕ_*n*_. **(B)** The optimally designed stimuli show specific patterns in the amplitudes *A*_*n*_ and the phases ϕ_*n*_. The rightmost column is a vector representation of *A*_*n*_ and ϕ_*n*_ using complex number *x* + *iy* = *A*_*n*_exp(*iϕ*_*n*_).

**Figure 5:**
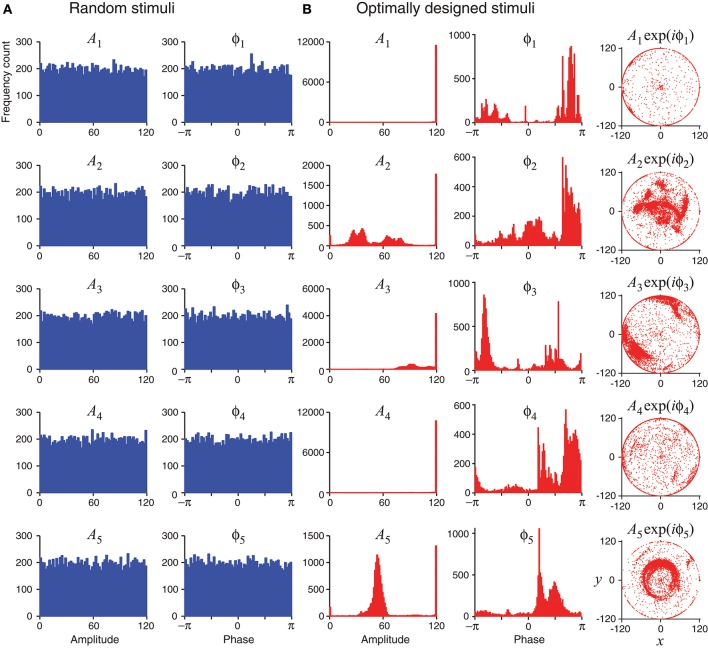
Statistics of random stimuli and optimally designed stimuli, showing distributions of their Fourier amplitudes *A*_1_, …, *A*_5_ and phases ϕ_1_, …ϕ_5_. Each subplot is based on 12,000 stimuli, either random or optimally designed. **(A)** Random stimuli were generated by choosing their Fourier amplitudes and phases randomly from a uniform distribution. **(B)** The optimally designed stimuli showed some structures in the distributions of the Fourier amplitudes and phases. The rightmost column is a vector representation of *A*_*n*_ and ϕ_*n*_ using complex number *x* + *iy* = *A*_*n*_exp(*iϕ*_*n*_).

Unlike random stimuli generated by Fourier series with random amplitudes and phases, the optimally designed stimuli seemed to have some inherent structures, which can be observed in the stimulus sequence generated one by one in a simulated experiment (Figure 4) as well as in the histograms obtained from repeated experiments (Figure 5). The appearance of oscillations in the sequence of optimally designed stimuli in Figure 4B is probably due to the spontaneous switching by the optimal design algorithm to probe different parameters (DiMattina and Zhang, 2011). The random stimuli were used as a control in our study. Their amplitudes *A*_*n*_ and the phases ϕ_*n*_ showed flat distributions in the histograms (Figure 5A). This is expected because the stimuli were generated by randomly drawing the amplitudes and phases from a uniform distribution. By contrast, the amplitudes *A*_*n*_ and the phases ϕ_*n*_ of the optimally designed stimuli were distributed in more complex manners (Figure 5B). For instance, the distribution of amplitude *A*_5_ (bottom panel) had a major peak in the middle, besides additional concentrations at either the lower bound or the upper bound. The lower and upper bounds mean the minimum and the maximum energy allowed for each Fourier component. The distributions of the amplitudes showed a tendency to cluster at the upper bound, which occurred in about half of the cases; the amplitudes lied between the lower and upper bounds also in about half of the cases, while the lower bound was reached in less than 2% of the cases. Our Fisher information maximization procedure yielded phase parameters with specific distributions. In fact, none of the phase parameters (rightmost column in Figure 5B) had a flat distribution; instead, they all seemed to have distributions with multiple peaks, indicating that the phases were not irrelevant variables for the optimal design.

The tendency for optimally designed stimuli to cluster around the topological boundary of the set of allowable stimuli was reported for feedforward network models (DiMattina and Zhang, 2011). In that situation, both the feedforward network and the stimuli were stationary without involving time. Here we found similar topological boundary property for time-varying stimuli in a dynamic recurrent network. We suspect there might exist deeper mathematical reasons underlying this phenomenon although a full and rigorous analysis is beyond the scope of this paper.

### 3.3. Maximum-Likelihood Estimates of Network Parameters

Given a dataset consisting of stimulus-response pairs, we can always use maximum-likelihood estimation to fit the network model to the data to recover all the parameters. Maximum-likelihood estimation is known to be asymptotically efficient in the limit of large data size, in the sense that the estimation is asymptotically unbiased (i.e, average of the estimates approaches the true value) and has minimal variance (i.e., the variance of the estimates approaches the Cramér-Rao lower bound).

The accuracies of the maximum-likelihood estimates from the optimally designed stimuli, as well as from the random stimuli, are shown in Table 3. Both types of stimuli yielded reasonably accurate estimates of the network parameters although some parameters appeared harder to estimate than others. The maximum-likelihood estimates based on optimally designed stimuli were closer to the true values, on average, than that based on random stimuli. Furthermore, the standard deviations of the estimates based on optimally designed stimuli were also smaller, indicating more consistent estimates in repeated trials.

**Figure 6:**
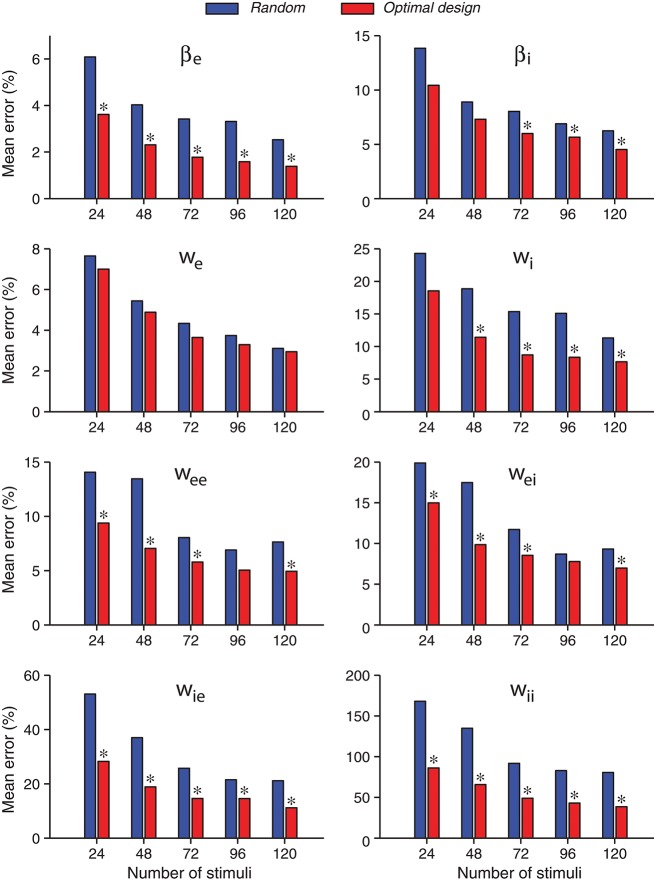
Errors of individual network parameters obtained by maximum likelihood estimation tended to decrease as the number of stimuli increased. Compared with the random stimuli, the optimally design stimuli yielded smaller errors for all network parameters in all the cases tested, although not all differences reached statistical significance. An asterisk (^*^) at the top of an optimal design bar indicates that the difference from the neighboring bar for random stimuli was statistically significant at the level *p* < 0.05 in the Wilcoxon rank-sum test. The mean error was calculated as the absolute value of the relative percentage of the difference between the estimate and the true value, averaged over 100 repeated trials.

A further comparison of the accuracies of individual parameter estimates is shown in Figure 6. As expected, the estimates of all parameters tended to be more accurate when the number of stimuli increased, both for the random stimuli and for the optimally designed stimuli. For every individual parameter, the error for the optimally designed stimuli was always smaller than that for the random stimuli, across all total numbers of stimuli we tested. The statistical significance of the differences was evaluated by Wilcoxon rank-sum test (Mann and Whitney, 1947; Gibbons and Chakraborti, 2011; Hollander et al., 2014). Most of the differences reached statistical significance at *p* = 0.05, as indicated by the asterisk (^*^) above the bars in Figure 6. That is, in all cases with an asterisk, the estimates from optimal design was significantly better than that from random stimuli.

With the total number of stimuli *M* = 120 (the largest sample size in our tests in Figure 6), the *p*-values of Wilcoxon rank-sum test for all the parameters estimated by the two methods were as follows: $p\left(\beta_{\text{e}}\right)=4.5\times 10^{-5}$, $p\left(\beta_{\text{i}}\right)=9.1\times 10^{-3}$, *p*(*w*_e_) = 0.989, $p\left(w_{\text{i}}\right)=6.9\times 10^{-3}$, $p\left(w_{\text{ee}}\right)=2.0\times 10^{-3}$, $p\left(w_{\text{ei}}\right)=5.6\times 10^{-3}$, $p\left(w_{\text{ie}}\right)=2.2\times 10^{-5}$, and $p\left(w_{\text{ii}}\right)=2.9\times 10^{-8}$. Thus the optimally design stimuli yielded significantly more accurate estimates than the random stimuli for 7 out of 8 parameters at the significance level *p* = 0.05. The only exception is the third parameter, *w*_e_, which was not significant regardless of the sample size *M* (Figure 6). This parameter was among the easiest to estimate because it had low errors relative to the true value for both methods (Table 3 and Figure 6). Although the optimal design method performed slightly better across all sample sizes, the small differences between the two methods did not reach statistical significance for our sample sizes (Figure 6). The error of parameter *w*_e_ might also be associated with the parameter confounding phenomenon, which will be discussed in section 3.5. The most significant difference, or the smallest *p*-value, was achieved for the parameter *w*_ii_, which was the hardest parameter to estimate because it had the largest errors relative to the true value for both methods (Table 3 and Figure 6). Parameter *w*_ii_ tended to have larger errors because the data recording was performed directly on the excitatory unit whose activity was affected only indirectly by the inhibitory unit self-connection weight. For the parameter which was the most difficult to estimate, the advantage of the optimal design over random stimuli was the most significant.

**Figure 7:**
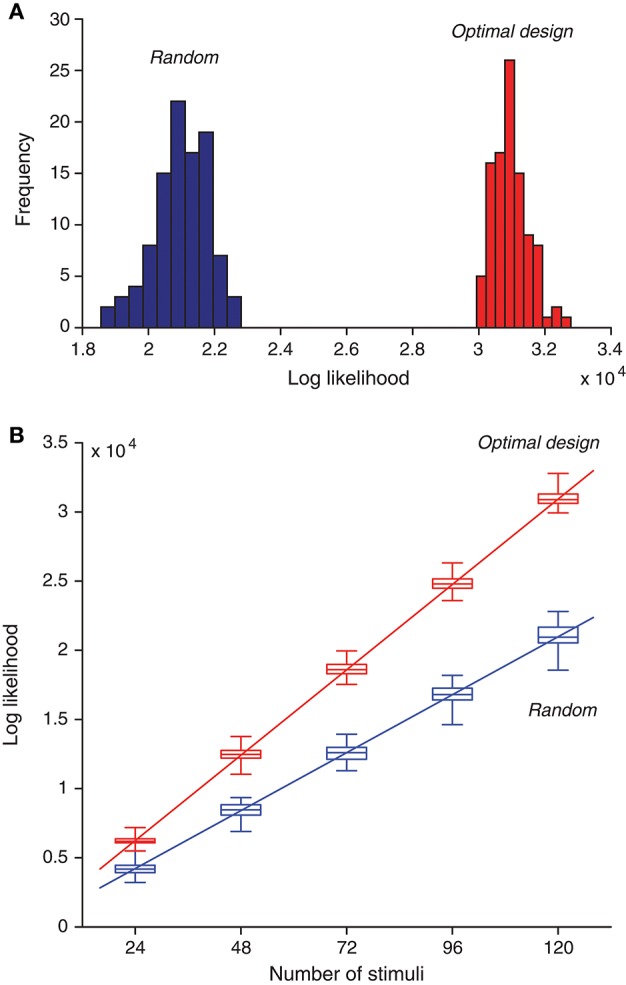
Optimally designed stimuli yield greater likelihood values for maximum-likelihood parameter estimation compared to random stimuli. **(A)** Histogram of optimized likelihood values for 100 trials with random stimuli is compared with that for 100 trails with optimally designed stimuli. Note that even the best value from the random trials was much worse than the worst value from the optimal design trials. Here each trial contained a sequence of 120 stimuli, generated either randomly or by optimal design. Each likelihood value was obtained by maximizing the likelihood function in Equation (11) using the response data elicited by all 120 stimuli. **(B)** As the number of stimuli increased, the likelihood function also increased, following an approximate linear relationship. The optimal design yielded better likelihood values than random stimuli regardless of the number of stimuli. The boxplot shows 25%, 50% (median), and 75% percentiles, with the whiskers outside of the boxes indicating the minimum and maximum values. The straight lines were obtained by linear regression on the median values [Equations (41) and (42)].

Besides means and standard deviations, we now examine the actual distributions of the likelihood values for the two types of estimates. We found that maximum likelihood obtained from the optimally design stimuli was typically much better than that obtained from the random stimuli (Figure 7A). A proper comparison should be based on the same number of stimuli. because for each estimation method, the likelihood value increased with the number of stimuli (Figure 7B), For a given number of stimuli, the optimally designed stimuli always yielded much greater likelihood value than the random stimuli. The minimum difference between the two sets of likelihood values (i.e., between the maximum from the random stimulus samples and the minimum from the optimal design samples) was typically at least two times greater than the standard deviation of either estimate except for the case with 24 samples (*M* = 24 with *N*_itr_ = 3). Even in this case, this violation occurred only for one outlier data point.

As expected from the log likelihood formula (11), the log likelihood value should increase approximately linearly with the number of stimuli, *M*, assuming that each stimulus, on average, contributes about the same to the log likelihood function. Although this assumption is not exactly true in further analysis, it is a good enough approximation that is compatible with the linear relationship. The linear relationship was confirmed by the numerical results in Figure 7B. Based on the regression lines, the values of the log likelihood have the following empirical formulas:

(41) $$\begin{array}{l} l_{\text{opt}}=257.1M_{\text{opt}}+76.2 \end{array}$$

for *M*_opt_ optimally designed stimuli, and

(42) $$\begin{array}{l} l_{\text{rand}}=174.5M_{\text{rand}}+31.1 \end{array}$$

for *M*_rand_ random stimuli. Both regression lines path through the origin point (0, 0) approximately (Figure 7B). This means that the ratio of the log likelihood values in the two cases is approximately a constant, as long as the two methods have the same number of stimuli. That is, when *M*_opt_ = *M*_rand_, it follows from Equations (41) and (42) that the log likelihood ratio is approximately:

(43) $$\begin{array}{l} \frac{l_{\text{opt}}}{l_{\text{rand}}}\approx \frac{257.1}{174.5}=1.47 \end{array}$$

where the small constant terms in Equations (41) and (42) are ignored. In other words, the two lines have a slope ratio of approximately 1.47. There is another way to look at the empirical formulas. For achieving the same level of likelihood value (*l*_opt_ = *l*_rand_), it follows from Equations (41) and (42) that

(44) $$\begin{array}{l} \frac{M_{\text{opt}}}{M_{\text{rand}}}\approx \frac{174.5}{257.1}=0.68 \end{array}$$

where once again the small constant terms in Equations (41) and (42) are ignored. Thus the optimal design saves the number of stimuli by about 32% or 1/3. Although this saving seems moderate, it may become useful in situations where it is extremely valuable to keep the duration of an experiment as short as possible.

### 3.4. Problem of Local Maxima During Optimization

We used optimization in two places: maximum likelihood estimation of parameters, and optimal design of stimuli. In the optimization process, local algorithms such as the constrained optimization function *fmincon* in Matlab need an initial guess to start the iterative procedure, but not all of the initial guesses will eventually converge to the true value. Generally speaking, as the number of initial guesses increases, it becomes more and more likely for one to find the global optimum rather get stuck in a local optimum. In the following, we perform an analysis to assess how likely our optimization processes were able to find the global optimum in repeated trials.

We emphasize that there is no need to always find the absolutely best solution in the actual optimization process. A good enough solution is probably good enough. Our purpose in this section is to perform a simple test to assess how hard our optimization problem really is. In repeated runs if all solutions end up to be approximately the same, it means that the optimization problem is easy, probably with a single global optimum, which makes repeated runs unnecessary. On the other hand, if there are many local optima, it is harder to hit on the best solution in a single run, and repeated runs become useful. In this situation it is also helpful to allow multimodal distribution of the parameter estimates so as to discourage early commitment to incorrect parameter values (see section 4).

The basic idea of our test is very simple. Let *p* be the probability of finding the “best” solution (tentative global optimum) in an individual run with a random initial guess. Suppose there are *n* repeats or starts from different initial values. Then in *n* repeated runs, the probably that at least one run will lead to the “best” solution is

(45) $$\begin{array}{l} \text{Prob (“best”)}=1-{\left(1-p\right)}^{n} \end{array}$$

A rough estimate of probability *p* can be obtained in two different ways, as explained below.

First, we consider the optimization process for maximum-likelihood estimation of parameters. We estimated the value of Prob (“best”) in (45) by starting the optimization from different initial guesses and then checking the number of solutions which stay in a relative error bound of fraction η for each individual parameter with respect to the best solution with the highest likelihood value. In other words, to pass the test the following criterion should be satisfied for all individual parameters (θ_1_, ⋯ , θ_8_) in (8):

(46) $$\begin{array}{l} \frac{\left|{\hat{\theta }}_{i}-{\hat{\theta }}_{i}^{\text{best}}\right|}{\theta_{i}}\leq \eta \;\left(i\;=1,\cdots \;,8\right) \end{array}$$

where θ_*i*_ is the true value, ${\hat{\theta }}_{i}$ is the estimate from a particular run, ${\hat{\theta }}_{i}^{\text{best}}$ is the best solution having the highest likelihood value in the repeated runs, and η is a constant. For simplicity we used η = 10% in all simulations reported in this paper; in other words, the error tolerance was set to be one order of magnitude smaller than the true parameter value. One may choose other values as well. To make the criterion less arbitrary, one could use Fisher information to estimate the asymptotic value of the error. In (46) we used ${\hat{\theta }}_{i}^{\text{best}}$ instead of the true value θ_*i*_ in the numerator of because even the true global maximum of the likelihood function could still be substantially different from the true parameter value. If the above was satisfied for all θ_1_, ⋯ , θ_8_, this result was counted as one pass of the test. By counting this way, we obtained an estimate of Prob (“best”), which was then inserted into (45) to solve for the probability *p*. The final result was *p* ≈ 0.85, which was based on 200 runs with 10 multiple initial guesses per 20 different stimuli configurations, and each configuration had *M* = 120 stimuli. In conclusion, in our maximum-likelihood parameter estimation, we might have a high probability (*p* ≈ 0.85) of obtaining a global maximum of the likelihood function in a single run. It implies that to get a 99% correct rate we would only need *n* = 3 repeats of the optimization procedure.

Next, we consider the optimization process for optimal design of stimuli. This optimization problem turned out to be much harder than the maximum-likelihood optimization described above because there were more local optima. Recall that the Fisher Information measure *U*_*k*_(*x*, θ) is computed with respect to each parameter θ_*k*_ as shown in (23). We need to consider how *U*_*k*_(*x*, θ) is maximized by the stimulus parameters **x** = [*x*_1_, ⋯ , *x*_2*N*_] = [*A*_1_, ⋯ , *A*_*N*_, ϕ_1_, ⋯ , ϕ_*N*_] as given in (15). Like in the case of likelihood analysis, we used repeated runs to estimate Prob (“best”) first and then computed *p* from (45). Similar to (46), the criterion for passing the test was $|{\hat{x}}_{i}-{\hat{x}}_{i}^{\text{best}}|/{\hat{x}}_{i}^{\text{best}}\leq 10\%$ for all *i*, where ${\hat{x}}_{i}$ is the value of parameter *x*_*i*_ obtained by maximizing *U*_*k*_(**x**, θ) in a particular run, and ${\hat{x}}_{i}^{\text{best}}$ is the parameter value that yielded the largest value of *U*_*k*_(**x**, θ) in the repeated runs. Here ${\hat{x}}_{i}^{\text{best}}$ is used in the denominator as well as in the numerator because unlike (46), there was no such as concept as a true stimulus parameter value.

Our simulation indicated that the maximum of the Fisher Information measure *U*_*k*_(**x**, θ) tended to depend more sensitively on the stimulus Fourier amplitudes *A*_*n*_ than the stimulus Fourier phases ϕ_*n*_. This difference might be related to the fact that the optimized amplitudes had a tendency to cluster at the upper bound, as described in section 3.2. In the following analysis, the criterion of finding the best solution was applied to the amplitudes only. The largest probability of finding the best solution for the amplitudes was *p* = 0.86, which was obtained for *U*_1_(**x**, θ), corresponding to the parameter θ_1_ = β_e_. The smallest probability *p* = 0.175 was obtained for *U*_5_(**x**, θ), corresponding to the parameter θ_5_ = *w*_ee_. The second and third smallest values *p* = 0.275 and *p* = 0.35 were obtained for *U*_3_(**x**, θ) and *U*_6_(**x**, θ), corresponding to θ_3_ = *w*_e_, and θ_6_ = *w*_ei_, respectively. It is worth mentioning that those three parameters were confounded or had strong correlations with at least one other network parameter (see section 3.5). Even in the worst case (*p* = 0.175 for *U*_5_(**x**, θ)), one would find the best solutions for the amplitudes with a 0.99 probability after *n* = 23 repeated runs with random initial guesses for the optimization procedure.

In summary, repeated runs with random initial guesses greatly increased the chance of finding the global maximum, although in our simulations this method appeared more effective for maximum-likelihood parameter estimation than stimulus optimization which suffered much more from the local optima problem. Fortunately, for practical applications, the existence of many more local maxima in the stimulus optimization process might not present a big problem because even partially optimized stimuli would still be better than nonadaptive random stimuli. There is a tradeoff between the quality of the stimulus optimization and the computational time, which could be reduced further in the future by the increasing power of parallel computing because repeated runs are readily parallelizable.

### 3.5. Parameter Confounding

**Figure 8:**
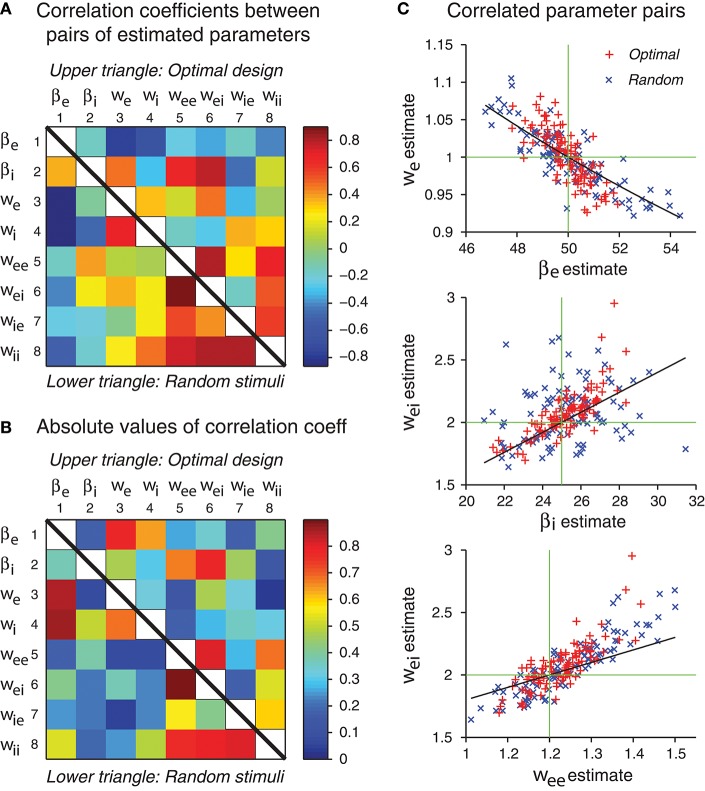
Some network parameters are approximately confounded, in the sense that a change of one parameter can be compensated by a proper change of another parameter, such that the stimulus-response relation of the whole network is kept approximately the same. **(A)** Correlation coefficient matrix of all possible pairs of network parameters obtained by maximum likelihood estimation from either optimal design data or random stimulus data. Since each matrix is symmetric, only a half needs to be shown. Here data from optimal design trials are shown in the upper triangle, whereas data from the random trials are shown in the lower triangle. Each data point was based on 100 repeated trials each containing a sequence of 120 stimuli. **(B)** Same data as in panel A, except that the absolute values are shown. **(C)** The three pairs of parameters with the highest correlation coefficients in the upper triangle in panel B are shown in the scatter plots. Data from both optimal design and random trials are shown. Black curves are theoretical predictions according to Equations (49), (53), and (54). Green crosshairs are centered at the true parameter values.

In our simulations, the errors of different parameter estimates were often correlated (Figure 8). Some of the correlations may be explained by parameter confounding. The basic idea is that different parameters may compensate each other such that the output of the network behaves essentially in the same way, even when the parameter values are configured differently. It is known that in individual neurons, different ion channels may be regulated such that diverse configurations may lead to neurons with similar neuronal behaviors in their electrical activation patterns (Prinz et al., 2004). Parameter confounding also exists at the network level, for example, in multilayer perceptrons (DiMattina and Zhang, 2010).

We will examine the original dynamical equations and demonstrate how approximate parameter confounding might arise. We emphasize that different parameters in the excitatory-inhibitory network are distinct and independent, and no strict confounding exists. All the parameter confounding problems considered here are approximate in nature.

Based on the analysis in Figures 8A,B, three pairs of parameters stand out with the strongest correlations. These pairs are (β_e_, *w*_e_), (β_i_, *w*_ei_) and (*w*_ee_, *w*_ei_). We will use the idea of parameter confounding to offer an intuitive heuristic explanation of why these three pairs tend to be correlated.

#### Example 1: Confounding of the Parameter Pair (β_e_, *w*_e_)

We first rewrite the dynamical Equations (3) and (4) in the following form:

(47) $$\begin{array}{l} \dot{V_{\text{e}}}=-\beta_{\text{e}}V_{\text{e}}+\beta_{\text{e}}\left\{w_{\text{ee}}g_{\text{e}}\left(V_{\text{e}}\right)-w_{\text{ei}}g_{\text{i}}\left(V_{\text{i}}\right)\right\}+\beta_{\text{e}}w_{\text{e}}I \end{array}$$

(48) $$\begin{array}{l} \dot{V_{\text{i}}}=-\beta_{\text{i}}V_{\text{i}}+\beta_{\text{i}}\left\{w_{\text{ie}}g_{\text{e}}\left(V_{\text{e}}\right)-w_{\text{ii}}g_{\text{i}}\left(V_{\text{i}}\right)\right\}+\beta_{\text{i}}w_{\text{i}}I \end{array}$$

The external stimulus *I* drives the first Equation (47) through the weight β_e_*w*_e_. If this product is the same, the drive would be the same, even though the individual parameters are different. For example, if β_e_ is increased by 25% from its true value while *w*_e_ is decreased by 20% from its true value, then their product β_e_*w*_e_ stays the same, so that the external input *I* provides the same drive to (47). Of course, any deviation from the true parameter values also leads to other differences elsewhere in the system. Therefore, the confounding relation is only approximate and not strict. This heuristic argument gives an empirical formula:

(49) $$\begin{array}{l} \beta_{\text{e}}w_{\text{e}}={\hat{\beta }}_{\text{e}}{ŵ}_{\text{e}} \end{array}$$

where β_e_ and *w*_e_ refer to the true values of these parameters, whereas ${\hat{\beta }}_{\text{e}}$ and ŵ_e_ refer to the estimated values.

#### Example 2: Confounding of the Parameter Pair (*w*_ei_, β_i_)

These two parameters appear separately in different equations, namely, *w*_ei_ appearing only in (47) while β_i_ appearing only in (48). To combine them, we need to consider the interaction of these two equations. To simplify the problem, we consider a linearised system around the equilibrium state:

(50) $$\begin{array}{l} \dot{V_{\text{e}}}=-\beta_{\text{e}}V_{\text{e}}+\beta_{\text{e}}\left\{w_{\text{ee}}k_{\text{e}}V_{\text{e}}-w_{\text{ei}}k_{\text{i}}V_{\text{i}}\right\}+\beta_{\text{e}}w_{\text{e}}I+C_{\text{e}} \end{array}$$

(51) $$\begin{array}{l} \dot{V_{\text{i}}}=-\beta_{\text{i}}V_{\text{i}}+\beta_{\text{i}}\left\{w_{\text{ie}}k_{\text{e}}V_{\text{e}}-w_{\text{ii}}k_{\text{i}}V_{\text{i}}\right\}+\beta_{\text{i}}w_{\text{i}}I+C_{\text{i}} \end{array}$$

where *k*_e_ and *k*_i_ are the slopes of the gain functions, and *C*_e_ and *C*_i_ are extra terms that depend on the equilibrium state and other parameters. Note that *V*_i_ appears in (50) only once, in the second term in the curly brackets. Since *V*_i_ also satisfies (51), we solve for *V*_i_ in terms of ${\dot{V}}_{\text{i}}$ from (51) and find a solution of the form: $V_{\text{i}}=c{\dot{V}}_{\text{i}}/\beta_{\text{i}}+a$ where *c* = −1/(1 + *w*_ii_*k*_i_) is a constant and *a* = (β_i_*w*_i_*I* + *C*_i_)/(β_i_(1 + *w*_ii_*k*_i_)) which depends on input *I*. Substitution into (50) eliminates *V*_i_, yielding an equation of the following form:

(52) $$\begin{array}{l} \beta_{\text{e}}^{-1}\dot{V_{\text{e}}}=\left(w_{\text{ee}}k_{\text{e}}-1\right)V_{\text{e}}-ck_{\text{i}}\left(w_{\text{ei}}/\beta_{\text{i}}\right)\dot{V_{\text{i}}}-k_{\text{i}}w_{\text{ei}}a+w_{\text{e}}I+C_{\text{e}}/\beta_{\text{e}} \end{array}$$

Note that the parameter combination *w*_ei_/β_i_ is a factor that scales how strongly ${\dot{V}}_{\text{i}}$ influences this equation. We use this combination as the basis for the following heuristic confounding relation:

(53) $$\begin{array}{l} w_{\text{ei}}/\beta_{\text{i}}={ŵ}_{\text{ei}}/{\hat{\beta }}_{\text{i}} \end{array}$$

#### Example 3: Confounding of the Parameter Pair (*w*_ee_, *w*_ei_)

These two parameters both appear in the curly brackets in (47). We have a heuristic confounding relation:

(54) $$\begin{array}{l} w_{\text{ee}}g_{\text{e}}\left({\overset{-}{V}}_{\text{e}}\right)-w_{\text{ei}}g_{\text{i}}\left({\overset{-}{V}}_{\text{i}}\right)={ŵ}_{\text{ee}}g_{\text{e}}\left({\overset{-}{V}}_{\text{e}}\right)-{ŵ}_{\text{ei}}g_{\text{i}}\left({\overset{-}{V}}_{\text{i}}\right) \end{array}$$

where ${\overset{-}{V}}_{\text{e}}$ and ${\overset{-}{V}}_{\text{i}}$ are the equilibrium states. If this equation is satisfied, we expect that the term in the curly brackets in (47) would be close to a constant [the right-hand side of (54)] whenever the state *V*_e_ and *V*_i_ are close to the equilibrium values. When the state variables vary freely, we expect this relation to hold only as a crude approximation.

The theoretical curves shown in Figure 8C are based on Equations (49), (53), and (54), and we emphasize that there is no free parameter. These three approximate confounding relations can qualitatively account for the data for optimally designed stimuli (Figure 8C). The data for random stimuli also appear to follow the same pattern (Figure 8C) although they seem to have more scattering than the optimal design counterpart. The theoretical slopes are somewhat smaller than the slopes of the empirical correlations, suggesting that the heuristic theory only accounts for a portion of the correlation. It is possible that the empirical correlations contain contributions from other parameters, beyond these three pairs and pair-wise correlations. The static multilayer perceptrons already show various types of parameter confounding behaviors (DiMattina and Zhang, 2010). The dynamic recurrent network models, which formally subsume the multilayer perceptrons as special equilibrium states, are expected to have more complicated behaviors of parameter confounding. In sum, our results indicate that the errors of different parameter estimates have a tendency to be correlated in specific manners, and some of the correlation patterns can be explained by an argument of approximate parameter confounding or compensation based on the differential equations of the underlying dynamics.

## 4. Discussion

We have implemented an adaptive design algorithm for generating dynamic stimuli that can efficiently probe a recurrent network with interacting excitatory and inhibitory units so as to optimize its parameter estimation. The network was a standard rate model with continuous dynamics which could reflect the average dynamics of a group of neurons with similar response properties. Since single-unit recordings in neurophysiological experiment yield spike train data, our optimal design process was based on artificial spike trains generated by an inhomogeneous Poisson process whose rate was determined by the continuous network state. Our simulated recording experiment was performed on the excitatory unit in the simplest excitatory-inhibitory model while the activity of the inhibitory unit was not directly known (Figure 1B). This situation was not unlike most neurophysiological experiments where there were always some relevant neurons whose activities were hidden from view but contributed indirectly to the recorded data. The time-varying stimuli were parameterized by a Fourier series whose parameters were determined by maximizing a utility function based on the Fisher information matrix; in other words, the stimuli were designed to elicit responses that would reveal the most information about the values of the network parameters. Each stimulus was fed to the network to elicit a Poisson spike train, and from the available stimulus-response data we inferred the values of all the network parameters, including the time constants and all the connection weights, by maximum-likelihood estimation, and the updated parameter values were then used to design the next stimulus, and so on, mimicking a closed-loop experiment (Benda et al., 2007; DiMattina and Zhang, 2013; Potter et al., 2014; El Hady, 2016). The optimization process was facilitated by knowing the derivatives of the network states with respect to the network parameters, and we found it convenient to compute the time evolutions of the derivatives by directly solving differential equations derived from the original system (Figure 3).

We confirmed that optimally designed stimuli elicited responses with much better likelihood values for parameter estimation than nonadaptive random stimuli (Figure 7), and every single parameter was recovered more accurately by the optimally designed stimuli (Figure 6). The statistical significance of the differences tended to be more pronounced for parameters that were harder to estimate with larger relative errors (section 3.3), and this observation underscores the value of the optimal design method for difficult estimation problems. Following the approximately linear relationship between the log likelihood and the number of stimuli (Equations 41, 42), we estimated that the optimal design would cut down the number of stimuli by about 1/3 to achieve the same level of parameter estimation accuracy as the nonadaptive random stimuli. Although this saving seems to be moderate, it still could potentially be important in real situations where time is extremely valuable, such as when recording time is seriously limited in experiments. We also noted that the Fourier amplitudes and phases of the optimally designed stimuli were not uniformly distributed but had specific features (Figure 5). Applying the features of optimally designed stimuli (e.g., the tendency for amplitude saturation) to bias the random stimuli could potentially lead to better parameter estimation (DiMattina and Zhang, 2011). In this paper we simply compare the optimal design against unbiased random stimuli because this is a fair comparison when one only knows the bounds of the parameters without any other information.

We found that the errors of different parameter estimates were correlated in specific ways, and some of the correlation patterns were rather predictable (Figure 8). We have derived heuristic formulas to account for some of the most prominent correlation patterns by an approximate theory of parameter confounding where the effect of changing one parameter was compensated by changing another parameter in a particular direction with an appropriate amount. This finding suggests that it would be useful in future studies to examine not only the errors of different parameter estimates but also their correlations, because they may reflect the underlying network connectivity and dynamics as predicted by the parameter confounding theory. It would be an interesting future research topic to study how to take advantage of the error correlation phenomenon to further improve the optimal design method.

Our stimulus design is based on maximizing an objective function or utility function, which may contain multiple local maxima. The existence of these local maxima makes it harder to find the global maximum. One related problem is early commitment to incorrect parameters during the iterative procedure. If the initial parameter estimates happen to be very wrong, then subsequent iterations may push the parameter estimation further down the wrong path, eventually getting stuck in an incorrect local maximum of the objective function. To assess how likely this may happen, we run the optimization procedure repeatedly with randomly chosen initial parameters and examined how often the final parameter estimates ended up around the putative global maximum (section 3.4). Although the problem of local maxima appeared manageable for the simple model studied in this paper, we expect that the problem may become more serious as model complexity increases. To better address the problem of early commitment to incorrect parameters, we should extend the theoretical framework to allow a potentially multimodal posterior distribution of the parameter values rather than only keeping the current best estimates. In a preliminary experiment of stimulus optimal design for auditory cortical neurons (Feng et al., 2012; Feng, 2013), four independent sets of parameter estimates were maintained throughout the sequential procedure so that a set of seemingly bad estimates at the initial stage could still have a chance to win the competition at a later time. In this work the stimuli as well as the model were stationary rather than time-varying. In the future it would be useful to develop our method for time-varying stimuli by incorporating a potentially multimodal posterior distribution of the parameters.

Speed of computation is an important issue when applying optimal design method to a real neurophysiological experiments. There is a basic tradeoff between the computational time and the quality of optimization in the optimal design. In our simulation, the optimization of a single 3-s long stimulus took about 15 min on a single PC with a 6-core Intel Core i7 processor. The number of steps required to converge to an optimum depended on multiple factors such as the current values of the network parameter estimates, the values of the objective function and the gradients, constraint violation, and step size. The optimization of the maximum likelihood parameter estimation had an additional problem: it tended to slow down as the spikes accumulate [see Equation (11)] leading to an objective function with gradually increasing complexity. An average value for the observed duration of the maximum likelihood optimization was about 38 min. As a result, optimization of one stimulus and subsequent maximum likelihood estimation took approximately 1 h. So one complete run of a simulated experiment with *M* = 120 optimally design stimuli took over 100 h.

To bring down the computation time for each 3-s stimulus to less than 3 s as required for real online experiments, we would need a 10^3^-fold speedup. There are several ways to speed up the computation although we did not try to implement them here since our focus in this paper was on offline simulation to prove the concept. In our simulation we integrated the equations using a time step of 0.001-s, which could be increased to a level as high as 0.01-s to achieve a 10-fold saving of computation steps. If an efficient and stable numerical differentiation algorithm can be employed, another optimality criterion such as *D* or *E* optimality could be used in the computation of the Fisher Information metric which might help reduce the number of steps. Knowing the fact that the optimal stimulus amplitudes tend to reach the upper boundary, with a certain probability we could set the amplitudes directly to the boundary as the starting point for optimization. The optimization algorithms could be further fine-tuned and streamlined to save time. It is also possible to save time by early stopping of the optimization process and not optimizing the parameters completely. Taking into account of all these factors considered above, we could conceivably achieve a speedup factor of 10 or more.

Several key computational procedures are highly parallelizable, such as multiple initial guesses for optimization. The stimuli could also be generated in parallel on multiple, independent processors as a block instead of one by one on a single processor. Generating the stimuli sequentially on multiple processors using past response data as if they were moving on an assembly line could also save time by a factor of 10 or more. Employment of larger cluster computing systems with faster processors and a large number of compute cores could potentially speed up the computation but this option is very expensive. A much more economical alternative is to take advantage of GPU computing which has been widely employed in the field of machine learning. If we could achieve a speedup factor of 10 or more by parallel computing and block design, the speed of our optimal design method would start to become relevant for real experiments.

Although the simulations reported here were focused on the simplest model which was adequate for our main conclusions within the scope of this paper, we emphasize that our method is readily generalizable to more complex network models. It should be straightforward to extend our derivations of the utility functions and the derivative equations to more complex networks with rate-based dynamics (Equation 1) because the method itself does not have an inherent limitation in this regard. We noted that even for such a simple network, some parameters, especially those related to the inhibitory unit which was not directly observed, were already quite hard to estimate, and the relative errors remained sizable even with the help of optimal design which worked significantly better than nonadaptive random stimuli. This result confirms that network parameter estimation is intrinsically a hard problem even for a simple network. For complex networks with more free parameters, it is reasonable to expect that the parameter estimation problem could become much harder. For models with more complex dynamics than the rate model, such as conductance-based models, the optimal design method needs to be adapted. The key modification would be the likelihood function of the spike train (Equation 11), which depends crucially on how noise is introduced into the model. Our method becomes irrelevant if the situation involves only deterministic spike trains. On the other hand, if the spikes can be well approximated by an inhomogeneous Poisson process, then our method could still be useful. Moreover, the method of gradient computation by solving differential equations is still a useful strategy because in all cases considered above the dynamics can always be described by a system of ordinary differential equations.

Advances in the technology for simultaneous recording from many neurons could increase the amount of information available about the underlying networks (Buzsáki, 2004; Cai et al., 2016; Jun et al., 2017) and thus potentially alleviate the parameter estimation problem in future online experiments. As mentioned before, optimal design methods have already been used in experiments on auditory neurons in the inferior colliculus (Dekel, 2012; Tam, 2012) and the auditory cortex (Feng et al., 2012; Feng, 2013). Those experiments were based on feedforward networks without temporal dynamics, and the spectral amplitudes of stationary sound stimuli were optimized to improve parameter estimation. The dynamic network models considered in this paper could be used to accommodate the transient response properties of auditory neurons such as the stereotypical time course with both transient and sustained response components (de la Rocha et al., 2008) (see also Figure 2). The computational study reported in this paper presents a necessary first step for extending the optimal design method to this type of dynamic network models for sensory neurons. In future studies it would be useful to apply the optimal design method to networks with a larger number of neurons in situations where the response data are collected simultaneously from multiple neurons, including both excitatory neurons and inhibitory neurons. More complex stimulus structures could be utilized. Besides the estimation of network parameters such as connection weights and the time constants, one may also estimate intrinsic parameters such as firing thresholds and slopes. Additional realistic details such as plasticity and adaptation could also be included. Although these generalizations require more computational power, the steady increase of accessible computational capacities over the years especially in parallel computing should make the optimal design method increasingly more feasible for real online experiments.

## Author Contributions

RD: performed the computations, prepared the tables, and wrote the first draft of manuscript. KZ: contributed to the theoretical development, polished up the figures, and finalized of the manuscript.

### Conflict of Interest Statement

The authors declare that the research was conducted in the absence of any commercial or financial relationships that could be construed as a potential conflict of interest.
